# Loss of pyrethroid resistance in newly established laboratory colonies of *Aedes aegypti*

**DOI:** 10.1371/journal.pntd.0007753

**Published:** 2020-03-16

**Authors:** Farah Z. Vera-Maloof, Karla Saavedra-Rodriguez, Rosa P. Penilla-Navarro, Americo D. Rodriguez-Ramirez, Felipe Dzul, Pablo Manrique-Saide, William C. Black

**Affiliations:** 1 Colorado State University, College of Veterinary Medicine and Biomedical Sciences, Department of Microbiology, Immunology and Pathology, Arthropod Borne and Infectious Diseases Laboratory, Campus Delivery, Fort Collins, Colorado, United States of America; 2 Centro Regional de Investigación en Salud Pública, Instituto Nacional de Salud Pública, Colonia Centro, Tapachula, Chiapas, México; 3 Centro Nacional de Programas Preventivos y Control de Enfermedades, Benjamín Franklin, Escandón, Ciudad de México, México; 4 Universidad Autonoma de Yucatan, México, Parque Santa Lucia, Centro, Mérida, Yucatan, Mexico; Centers for Disease Control and Prevention, UNITED STATES

## Abstract

**Background:**

Resistance to pyrethroid insecticides in *Aedes aegypti* has become widespread after almost two decades of the frequent use of these pesticides to reduce arbovirus transmission. Despite this resistance, pyrethroids continue to be used because they are relatively inexpensive and have low human toxicity. Resistance management has been proposed as a way to retain the use of pyrethroids in natural populations. A key component of resistance management is the assumption that negative fitness is associated with resistance alleles such that resistance alleles will decline in frequency when the insecticides are removed. At least three studies in *Ae*. *aegypti* have demonstrated a decrease in pyrethroid resistance once the insecticide has been removed.

**Methods/Principal findings:**

The present study aims to evaluate variation in the loss of pyrethroid resistance among newly established laboratory populations of *Ae*. *aegypti* from Mexico. Eight field collections were maintained for up to eight generations, and we recorded changes in the frequencies of the mutations at the V1,016I locus and at the F1,534C locus in the voltage-gated sodium channel gene (*VGSC*). I1,016 and C1,534 confer resistance. We also examined resistance ratios (RR) with type 1 and 2 pyrethroids.

**Conclusions/Significance:**

We demonstrate that, in general, the frequency of the *Ae*. *aegypti* pyrethroid-resistance alleles I1,016 and C1,534 decline when they are freed from pyrethroid pressure in the laboratory. However, the pattern of decline is strain dependent. In agreement with earlier studies, the RR was positively correlated with the frequencies of the resistance allele I1,016 and showed significant protection against permethrin, and deltamethrin, whereas F1,534C showed protection against permethrin but not against deltamethrin.

## Author summary

The author is interested in the evolution of genes that confer resistance to insecticides, especially when this evolution affects the binding of insecticides to their target sites. The Voltage-Gated Sodium Channel gene provides an excellent opportunity to understand how mutations at the target site(s) affect the evolution of resistance in many different pest insect species, including *Aedes aegypti*, which is the primary vector of the dengue, yellow fever, Zika and chikungunya viruses.

## Introduction

After almost two decades of frequent pyrethroid use for *Aedes aegypti* (L.) control, widespread resistance now exists [1, 2]. Despite this, pyrethroid use continues because these insecticides are relatively inexpensive and have low human toxicity. Resistance management has been proposed as a way to preserve the effectiveness of pyrethroids for *Ae*. *aegypti* control programs [2]. A key component of resistance management is the assumption that negative fitness is associated with resistance alleles such that the resistance alleles will decline in frequency when the insecticides are removed.

Laboratory strains of *Ae*. *aegypti* have shown a decrease in resistance once a pyrethroid is removed, thereby suggesting a fitness cost is associated with resistance. To date, three laboratory studies have evaluated the loss of pyrethroid resistance in *Ae*. *aegypti*. In Taiwan, a permethrin-resistant laboratory strain was maintained for 47 generations under permethrin pressure. Following 15 generations without exposure, a significant decrease was observed in the permethrin resistance ratio (RR) and resistance alleles in the Voltage-Gated Sodium Channel gene (VGSC) [3]. In Brazil, after 15 generations, the frequency of I1,016 decreased from 0.75 to 0.20 [4]. A study in Mexico showed a significant increase in the proportion of knocked-down mosquitoes ten generations after removal of pyrethroid exposure [5] but without a decrease in the frequency of I1,016 and C1,534 mutations in the VGSC. All three studies were done in laboratory cages.

The present study aims to evaluate the loss of pyrethroid resistance from eight collections of *Ae*. *aegypti* (six field collections from or near the city of Merida and two collections from Tapachula and Acapulco from southern Mexico). These collections were maintained without pyrethroid pressure for eight consecutive generations; during this time, we recorded changes in the frequencies of two mutations—VGSC I1,016 and C1,534—and analyzed the resistance ratios (RR) for permethrin (pyrethroid type 1) and deltamethrin (pyrethroid type 2).

## Materials and methods

### *Aedes aegypti* field populations

In 2014, larvae were collected from eight public sites in Mexico. These larvae were reared to adults and then identified as *Ae*. *aegypti*. The adult females were then blood fed (citrated sheep blood–Colorado Serum Co., Denver Colorado) using an artificial membrane feeder, and the eggs were collected for shipment. The GPS coordinates and name abbreviations of the collection sites appear in Table 1. Eggs from Yucatan were collected from three urban sites in Merida and from three villages near Merida. Two additional collections were taken from Tapachula and Acapulco, in the states of Chiapas and Guerrero, respectively.

**Table 1:** Collection sites by state and city, geographical coordinates, and site abbreviations.

State	City	Site	Latitude	Longitude	Abbreviation
**Guerrero**					
	Acapulco	Zapata	16.9049222	-99.8410944	**Acp**
**Chiapas**					** **
	Tapachula	Col. 5 de Febrero	14.9204944	-92.2593472	**Tap**
**Yucatan**					** **
	Merida	Fco. Montejo	21.0307194	-89.6463639	**Mer1**
		Plan Ayala	21.0135833	-89.6222222	**Mer2**
		U.H. Morelos	20.9420139	-89.5981556	**Mer3**
	Conkal	Center	21.0747917	-89.5199056	**Co**
	Dzitya	Center	21.0623278	-89.6746694	**Dz**
	Acanceh	Center	20.8126083	-89.4505611	**Ac**

### Establishment and maintenance of field populations

F_1_ eggs were sent to Colorado State University. Egg papers were placed in a container with 2 L of tap water to promote development and hatching. Larvae were fed 2 mL of 10% (w/v) liver powder solution every other day. We transferred pupae to cups inside plastic cages for adult emergence. Larvae and mosquitoes were maintained in an incubator at 27–28°C, 70–80% humidity and a photoperiod of 12 h light:12 h dark. Adults were fed with raisins and allowed access to tap water. Females were offered citrated sheep blood in artificial membrane feeders, every four days, to obtain eggs. Females laid their eggs on moistened filter papers. The eggs were allowed to mature for 48 h before being allowed to partially dry at room temperature; they were then stored in sealed plastic bags. Each collection was split at the F_1_ larval stage into three groups of 500 to act as biological replicates. In each subsequent generation, ≈250 adult ♀ and 250 adult ♂ were used to maintain each of the three replicates.

### Resistance ratio

We evaluated the changes in the resistance levels by determining the LC_50_ for permethrin and deltamethrin at each of the eight sites in the F_3_, F_5_, and F_8_ generations. Resistance ratios were obtained by dividing the LC_50_ calculated for each site by the LC_50_ calculated for the New Orleans *Ae*. *aegypti* (NO) susceptible reference strain. This colony was originally collected in New Orleans, Louisiana, by the Centers of Disease Control and Prevention and donated by Dr. William Brogdon. Our laboratory has maintained this colony since 2005 free of insecticide exposure. None of the knockdown resistance (kdr) alleles (I1,016 nor C1,534) are present in NO, and pyrethroid susceptibility is routinely confirmed by bottle bioassays. Commonly, the LC_50_ for permethrin ranges between 0.4 and 0.6 ug/bottle, and for deltamethrin, between 0.09 and 0.15 ug/bottle. We examined the relationship between the RR and the frequencies of the I1,016 and C1,534 alleles. Analyses were performed in the F_3_, F_6_, and F_8_ generations and then for all three generations combined. We used PROC CORR in SAS 9.4 to calculate Pearson’s correlation coefficient and to test for significance.

### Genotyping V1,016I and F1,534C

DNA was extracted at each generation (F_1_-F_8_) from individual mosquitoes by the salt extraction method [6] and resuspended in 180 μL of TE buffer. To identify allelic variation, we used the allele-specific polymerase chain reaction (asPCR), followed by generation of a melting curve (CFX-96 BioRad), to identify the genotypes [7–9]. In each of the eight generations, we analyzed three replicates of 50 adult mosquitoes (~25♀ and 25♂) for each of the eight collection sites. Sample sizes were kept intentionally large to minimize the founder effect and genetic drift.

### Allele frequencies and linkage disequilibrium analysis

We estimated allele frequencies in each of the eight generations from the genotypic frequencies (resistant allele frequency) = (2 x resistant homozygote + heterozygote) / (2 x sample size). Allele frequencies were compared among the replicates using a 2x3 contingency χ^2^ test. WinBUGS 2.0 [10] with 10^6^ iterations was used to calculate the 95% high-density intervals (HDI 95%) around the allele and genotype frequencies. We used ggplot2 in R-3.5.1 to graph the data. In addition, we used LINKDIS [11] and χ^2^ tests to calculate the pairwise linkage disequilibrium coefficients (R_ij_) between alleles at loci 1,016 and 1,534 [12] according to the following:

$$R_{ij}=\Delta_{ij}/\sqrt{((}pi(1-pi)+Ci)(pj(1-pj)+Cj))$$

where Ci = Hobs (i)–p_i_^2^, Hobs (i) is the observed frequency of the i homozygotes, and

$$\Delta_{ij}=\left(N/\left(N‐1\right)\right)\;\left(\left(T_{ij}/N\right)–2p_{i}p_{j}\right).$$

N is the sample size, and p_i_ and p_j_ are the frequencies of the alleles at locus i = 1,016 and locus j = 1,534, respectively. T_ij_ is the number of times that allele i and allele j occur in the same individual. A χ^2^ test was performed to determine if significant disequilibrium exists among all alleles at 1,016 and 1,534. The statistic was calculated and summed over all two-allele interactions as follows:

$$\chi^{2}{}_{\left[1\;d.f.\right]}=N\sum_{i}\sum_{j}\;(\Delta_{ij}{}^{2}/p_{i}p_{j})$$

## Results

### Resistance declines in absence of pyrethroids

**Fig 1:**
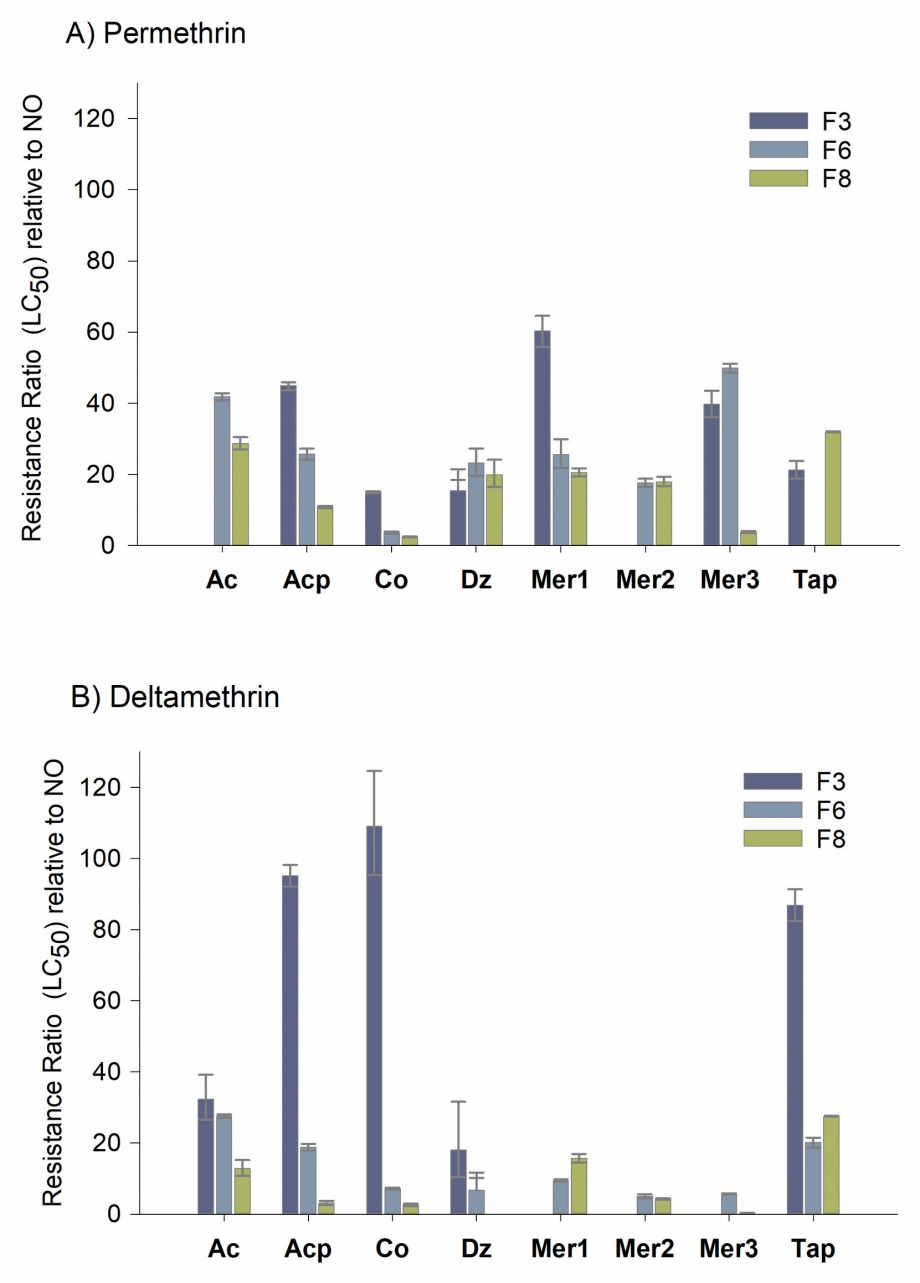
**A) Resistance to permethrin in generations F**_**3**_**, F**_**6**_ **and F**_**8**_ **in absence of insecticides.** Resistance ratios were calculated by dividing the LC_50_ of our sites by the LC_50_ determined for New Orleans. **B) Resistance to deltamethrin in generations F**_**3**_**, F**_**6**_ **and F**_**8**_ **in absence of insecticides.** Resistance ratios were calculated by dividing the LC_50_ of our sites by the LC_50_ determined for New Orleans.

Permethrin and deltamethrin LC_50_ was determined at generations F_3_, F_6_ and F_8_ (S1 Table). These values were compared against the NO susceptible colony to obtain the resistance ratios (RR). Fig 1 and S1 Table show the resistance ratios (RR) obtained for both pyrethroids at each of the generations evaluated. Permethrin resistance in the F_3_ varied from 15-fold to 60-fold in six of the eight sites (Fig 1A). The most resistant colony was Mer3 (60-fold) followed by Acp (45-fold) and Mer3 (39-fold). The remaining colonies had RR between 15-fold to 21-fold. At the F_6_ generation, RR ranged between 3-fold to 50-fold. The most resistant sites were Mer3 (50 fold) and Ac (42-fold). Acp, Dz, Mer1 and Mer2 had RR from 17-fold to 25-fold. At the F_8,_ permethrin RR varied from 2-fold to 28-fold. In general, permethrin RR declined after eight generations in five sites (Ac, Acp, Co, Mer1 and Mer3) whereas two sites did not show changes in RR (Dz and Tap).

Deltamethrin RR ranged from 18-fold to 108-fold in generation F_3_ (Fig 1B). The most resistant colonies were Co, Acp and Tap with RR higher than 86-fold. Ac and Dz had RR of 32-fold and 18-fold, respectively. Unfortunately, we did not have enough material to process Mer1, Mer2 and Mer3 at this generation. In generations F_6_ and F_8_, RR ranged from 3-fold to 27-fold. In general, we observed a decline in RR in five sites (Ac, Acp, Co, Dz and Tap). Mer1 and Mer 2 showed a slight decrease from the F_6_ to F_8_.

### Allele frequency of I1,016 declines in the absence of pyrethroids

**Fig 2:**
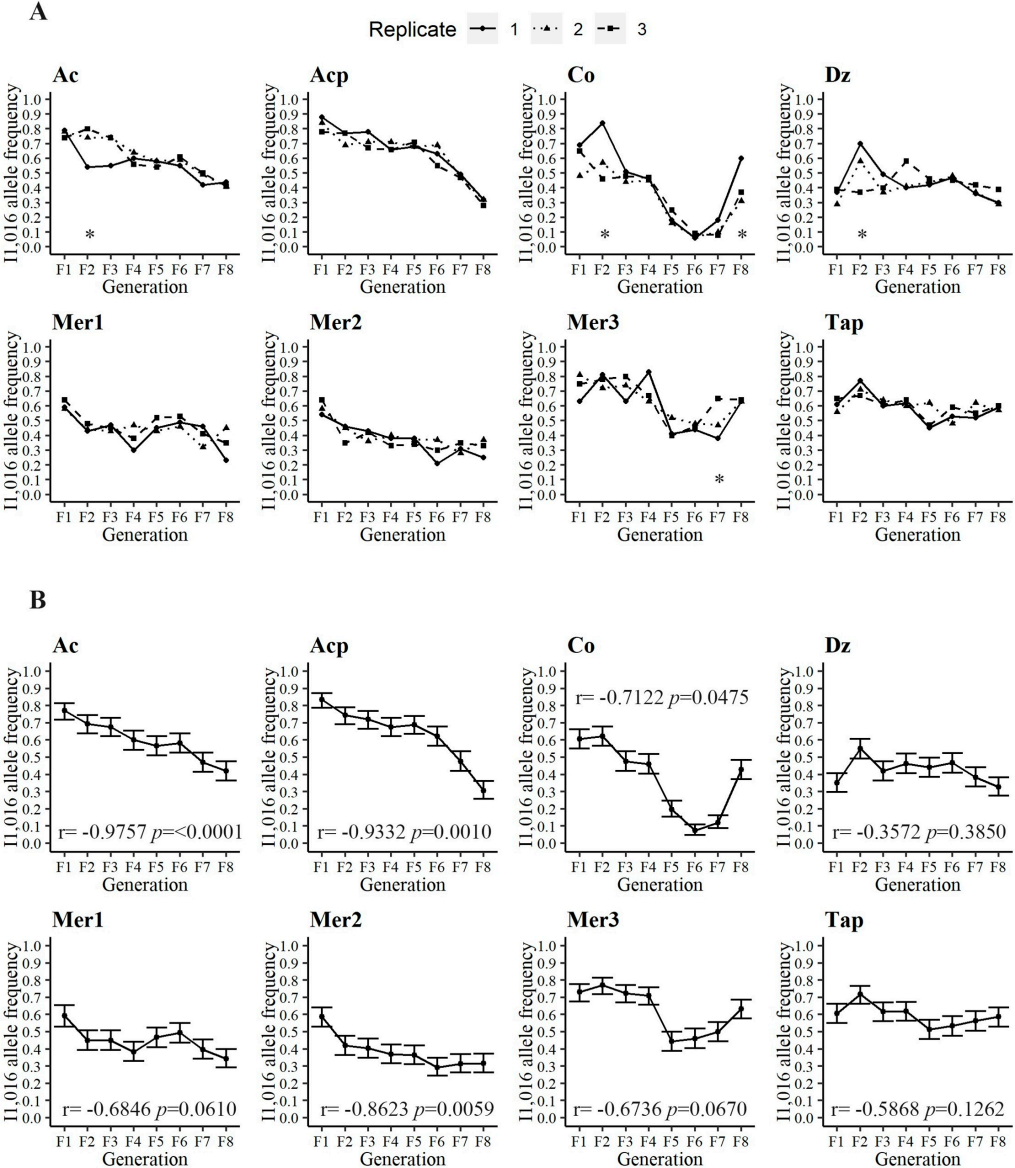
**A) Frequency of the resistant allele I1,016 in all three replicates in the eight different collections over eight generations.** Asterisks indicate a statistical difference among the replicates. **B) Mean frequency of the resistant allele I1,016 among all three replicates and the 95% high-density intervals (HDI 95**%). Pearson correlation coefficient between I1,016 frequencies and generation number and the associated significance appear at the bottom of each graph.

**Fig 3:**
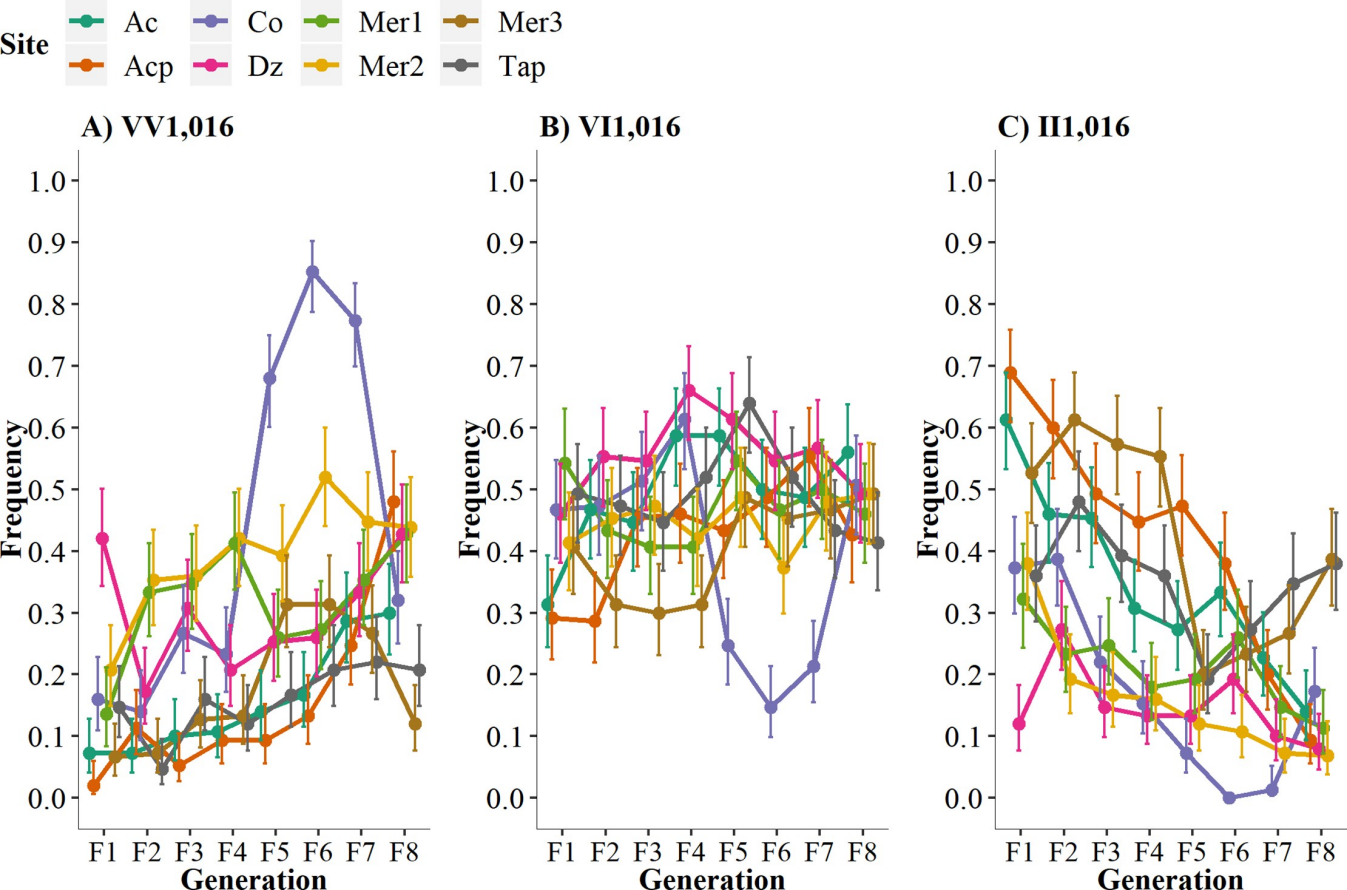
**A) Frequency of the resistant allele C1,534 in all three replicates in the eight different collections over eight generations**. An asterisk indicates statistical differences among the three replicates. **B) Mean frequency of the resistant allele C1,534 among all three replicates and the 95% high-density intervals (HDI 95**%). The Pearson correlation coefficient between the C1,534 frequencies and generation number and the associated significance appear at the bottom of each graph.

**Fig 4:**
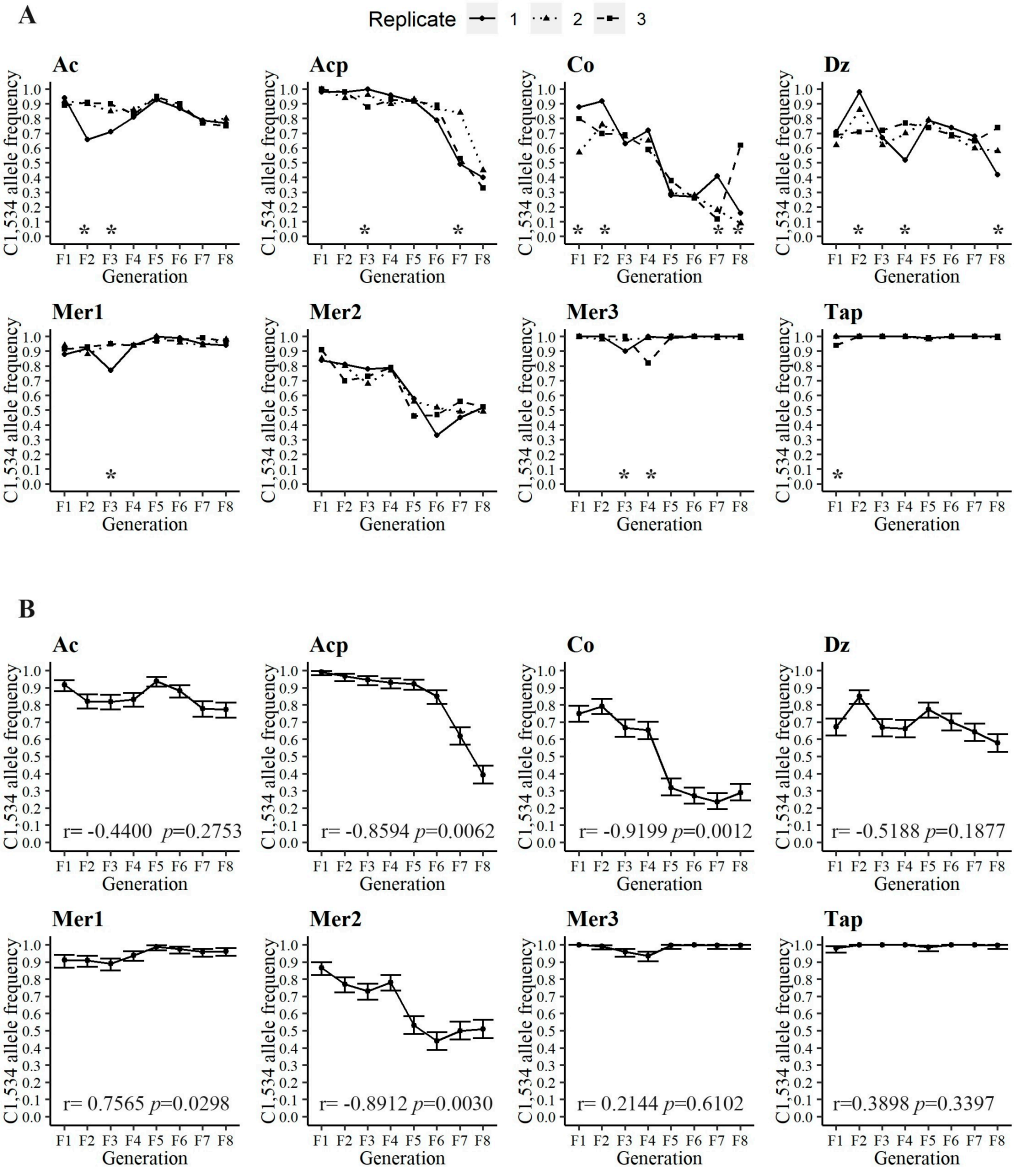
Frequency of genotypes at locus V1,016I over eight generations in the absence of pyrethroids. **A)** Susceptible-homozygote = VV_1,016_, B) Heterozygote = VI_1,016_, and C) Resistant-homozygote = II_1,016_.

**Fig 5:**
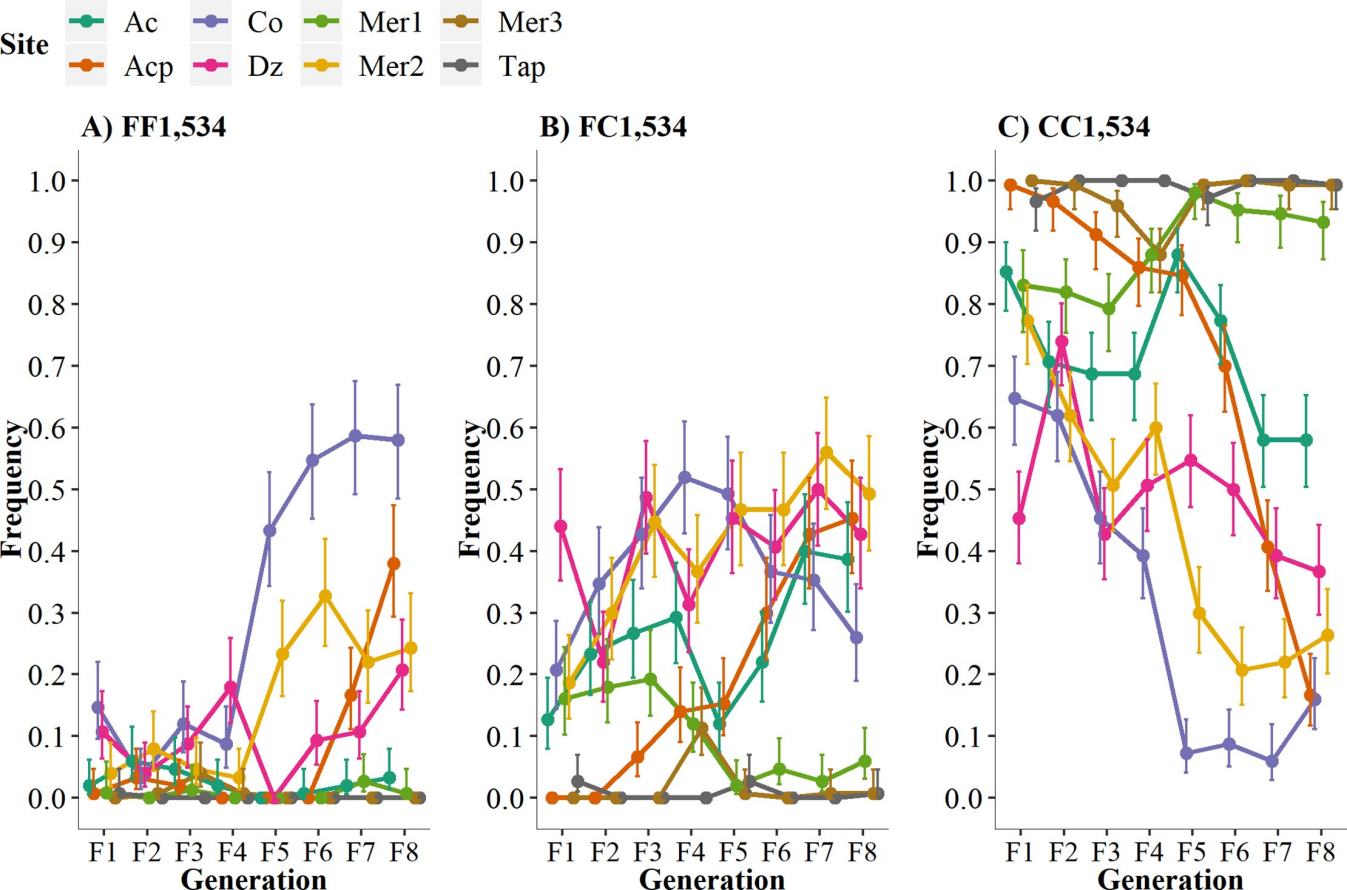
Frequencies of genotypes at locus F1,534C over eight generations in the absence of insecticides. Susceptible-homozygote = FF_1,534_, heterozygote = FC_1,534_, and resistant-homozygote = CC_1,534_.

We determined the V1,016I and C1,534 genotypes of 9,563 mosquitoes from eight sites in southern Mexico (Table 1) over eight generations in absence of pyrethroids (F_1_-F_8_). The genotype counts and allele frequencies appear in S2 and S3 Tables, respectively. Allele and genotype frequencies were analyzed separately for each locus. We show a general decline of the resistance alleles in Figs 2 and 4; and the different tendencies of each of the genotypes in Figs 3 and 5.

Fig 2A plots the I1,016 frequencies in all three replicates in the eight different collections over eight generations. Initial allele frequencies in the F_1_ varied from 0.35 in Dz to 0.83 in Acp, most of the sites had frequencies ranging between 0.59 to 0.73. In general, the I1,016 allele frequencies were statistically uniform among the three replicates, with five exceptions that are indicated with an asterisk (AcF_2_, CoF_2_, CoF_8_, DzF_2_, and Mer3F_7_) (Fig 2A). Fig 2B plots the mean I1,016 frequencies among all three replicates and the 95% high-density intervals (HDI 95%). The Pearson correlation coefficients between the I1,016 frequencies and the generation number and the associated significance appear at the bottom of each graph. The correlation between the I1,016 allele frequency and generation number was negative in each of the eight collections, though statistical significance varied from highly significant in four out of the eight sites (Ac, Acp, Co, and Mer2) (Fig 2B) to not significant in sites Dz, Mer1, and Tap. The frequency of I1,016 in Co, Mer3, and Tap declined initially and then, surprisingly, increased in the last 3–4 generations. In general, I1,016 declined in frequency over eight generations; however, the rate and pattern of the decline varied greatly among the collections.

### Genotype frequencies at 1,016 indicate lower fitness of the resistant-homozygote

To understand the fitness of each genotype, we examined the fate of the three genotypes separately over time (Fig 3). Because the knockdown resistance (kdr) is a recessive trait (only homozygote resistant genotypes survive exposure to pyrethroids), we expect a strong negative fitness cost for homozygote-resistant individuals (II_1,016_) in the absence of pyrethroids. We show in Fig 3C that a decline in II_1,016_ occurs over eight generations, and Table 2 shows negative correlation coefficients for all sites (*r* = -0.5452, P < 0.0001), confirming a negative fitness for the II_1,016._ Additionally, we show that the susceptible-homozygote or wild-type genotype (VV_1,016_) increased in frequency over the generations with positive correlation coefficients (*r* = 0.4497, *p* = 0.0002). This suggests that the susceptible homozygote genotype has greater fitness in the absence of insecticides. Interestingly, we did not observe changes in the frequency of the heterozygotes over time (*r* = 0.1462, P = 0.2489), indicating that no negative fitness cost occurs for VI_1,016_ in laboratory conditions. Therefore, the resistant allele will prevail in heterozygous individuals for several generations in the absence of insecticides.

**Table 2:** Pearson's correlation coefficient between the genotypes at locus V1,016I and generations in the absence of insecticides. Susceptible-homozygote = VV_1,016_, heterozygote = VI_1,016_, and resistant-homozygote = II_1,016_.

	VV_1,016_		VI_1,016_		II_1,016_	
Site	Pearson r	*P* value	Pearson r	*P* value	Pearson r	*P* value
**Ac**	0.9301	0.0008	0.6406	0.0870	-0.9418	0.0005
**Acp**	0.8156	0.0136	0.7455	0.0338	-0.9619	0.0001
**Co**	0.6499	0.0811	-0.4268	0.2916	-0.7886	0.0200
**Dz**	0.2371	0.5718	0.1178	0.7811	-0.4836	0.2247
**Mer1**	0.5472	0.1604	0.0404	0.9244	-0.8005	0.0170
**Mer2**	0.8062	0.0156	0.3131	0.4502	-0.8753	0.0044
**Mer3**	0.5830	0.1293	0.7033	0.0516	-0.7085	0.0492
**Tap**	0.7559	0.0300	-0.1720	0.6838	-0.3615	0.3789
**Across all**	0.4497	0.0002	0.1462	0.2489	-0.5452	<0.0001

### Allele frequency of C1,534 declines in the absence of pyrethroids

We obtained the genotypes and allele frequencies for F1,534C in each of the 9,563 mosquitoes for which the V1,016I genotype frequencies had been determined (S2 and S3 Tables). Fig 4A shows the resistant allele C1,534 frequencies in the eight collection sites over eight generations. Initial allele frequencies in the F_1_ ranged from 0.67 in Dz to 0.98 in Acp and Tap. The C1,534 frequencies differed between replicates in 15 of the 64 comparisons. The correlation coefficients between the C1,534 allele frequencies and generation number and their significance are at the base of each of the graphs in Fig 4B. The correlation coefficients between the C1,534 frequency and generation number were negative and significant in four out of the eight collection sites for the F_1_ to F_8_ generations (Acp, Co, Mer1, and Mer2) (Fig 4B). The correlation was not significant for sites Ac, Dz, Mer3, and Tap. In general, C1,534 declined in frequency over the eight generations; however, the rate and pattern of the decline varied greatly among the collections.

### Genotype frequencies at 1,534 indicate lower fitness of the resistant-homozygote

The genotype frequencies at F1,534C are provided in Fig 5, and the correlation coefficients between genotypes and generations are provided in Table 3. Five correlation coefficients for FF_1,534_ were positive, with two being significant; five of the correlation coefficients for FC_1,534_ were positive, with three being significant; and five of the CC_1,534_ were negative, with four being significant. Across all collection sites, a positive correlation (*r* = 0.3399, *p* = 0.006) existed between the homozygote susceptible FF_1,534_ and the generation number. The frequencies of FC_1,534_ did not change significantly over the generations (*r* = 0.2078, P = 0.0994), and as expected for a genotype that confers a lower fitness, the CC_1,534_ genotypic frequencies decreased significantly over the generations (*r* = -0.3024, P = 0.0152), suggesting a fitness cost associated with the resistant-homozygote genotype.

**Table 3:** Pearson's correlation coefficient between the genotypes at locus F1,534C and generations in the absence of insecticides. Susceptible-homozygote = FF_1,534_, heterozygote = FC_1,534_, and resistant-homozygote = CC_1,534_.

	FF_1,534_		FC_1,534_		CC_1,534_	
Site	Pearson r	*P* value	Pearson r	*P* value	Pearson r	*P* value
**Ac**	-0.363	0.3759	0.6537	0.0788	-0.5460	0.1615
**Acp**	0.6952	0.0556	0.9711	<0.0001	-0.9110	0.0016
**Co**	0.8953	0.0026	0.0528	0.9012	-0.8996	0.0023
**Dz**	0.3782	0.3555	0.3733	0.3624	-0.5175	0.189
**Mer1**	0.2729	0.5131	-0.8254	0.0116	0.7953	0.0183
**Mer2**	0.7997	0.0172	0.8748	0.0045	-0.9131	0.0015
**Mer3**	-0.3423	0.4066	-0.0164	0.9692	0.1308	0.7575
**Tap**	-0.5774	0.1340	-0.2701	0.5177	0.3309	0.4233
**Across all**	0.3399	0.006	0.2078	0.0994	-0.3024	0.0152

### Linkage disequilibrium

We performed pairwise linkage disequilibrium analyses between the alleles in V1,016I and F1,534C. Table 4 lists the linkage disequilibrium coefficients R_ij_, χ^2^, and the probability value obtained between pairwise loci. R_ij_ is distributed from -1.00 (mutations occur on opposite chromosomes—*trans*) to 0.00 (mutations occur independently) to 1.00 (both mutations on the same chromosome—*cis*); therefore, R_ij_ provides a standardized measure of the disequilibrium.

**Table 4:** Linkage disequilibrium coefficients between loci 1,016 and 1,534 over eight generations in absence of pyrethroids.

		1,016–1,534					1,016–1,534		
Site	Generation	R_ij_	χ^2^	*P value*	Site	Generation	R_ij_	χ^2^	*P value*
**Acp**					**Dz**				
	F2	0.40262	24.32	0.0001		F1	0.47671	34.09	0.0001
** **	F3	0.40043	24.05	0.0001		F2	0.44321	29.46	0.0001
	F4	0.40285	24.34	0.0001		F3	0.50702	38.56	0.0001
** **	F5	0.53718	43.28	0.0001		F4	0.32897	16.23	0.0001
	F6	0.52471	41.3	0.0001		F5	0.35	18.38	0.0001
** **	F7	0.65434	64.22	0.0001		F6	0.55227	45.75	0.0001
	F8	0.85375	109.33	0.0001		F7	0.47286	33.54	0.0001
**Tap**						F8	0.52979	42.1	0.0001
	F1	0.18597	5.19	0.0227	**Co**				
**Mer1**						F1	0.63877	61.2	0.0001
	F2	0.3114	14.55	0.0001		F2	0.46501	32.43	0.0001
** **	F3	0.21116	6.69	0.0097		F3	0.61457	56.65	0.0001
	F4	0.30385	13.85	0.0002		F4	0.67762	68.88	0.0001
** **	F7	0.20948	6.58	0.0103		F5	0.67333	68.01	0.0001
	F8	0.23208	8.08	0.0045		F6	0.38327	22.03	0.0001
**Mer2**						F7	0.6995	73.39	0.0001
	F1	0.44441	29.63	0.0001		F8	0.15305	3.51	0.0609
** **	F2	0.47953	34.49	0.0001	**Ac**				
	F3	0.4159	25.95	0.0001		F1	0.54278	44.19	0.0001
** **	F4	0.30371	13.84	0.0002		F2	0.59628	53.33	0.0001
	F5	0.74874	84.09	0.0001		F3	0.68619	70.63	0.0001
** **	F6	0.7115	75.93	0.0001		F4	0.49335	36.51	0.0001
	F7	0.6068	55.23	0.0001		F5	0.30868	14.29	0.0002
** **	F8	0.66266	63.23	0.0001		F6	0.33043	16.38	0.0001
**Mer3**						F7	0.59792	53.63	0.0001
** **	F2	0.20207	6.12	0.0133		F8	0.4856	35.37	0.0001
	F3	0.42021	26.49	0.0001					

Alleles segregated in 57 of the 64 collections (Table 4). Forty-seven of the 57 collections exhibited significant linkage disequilibrium, with the R_ij_ values ranging between 0.15 and 0.85 among the collections. This result suggests that the resistance alleles I1,016 and C1,534 occur together more often than expected by independent, random segregation. In general, and in agreement with earlier studies [12, 13], alleles in V1,016I and F1,534C were in linkage disequilibrium.

**Fig 6:**
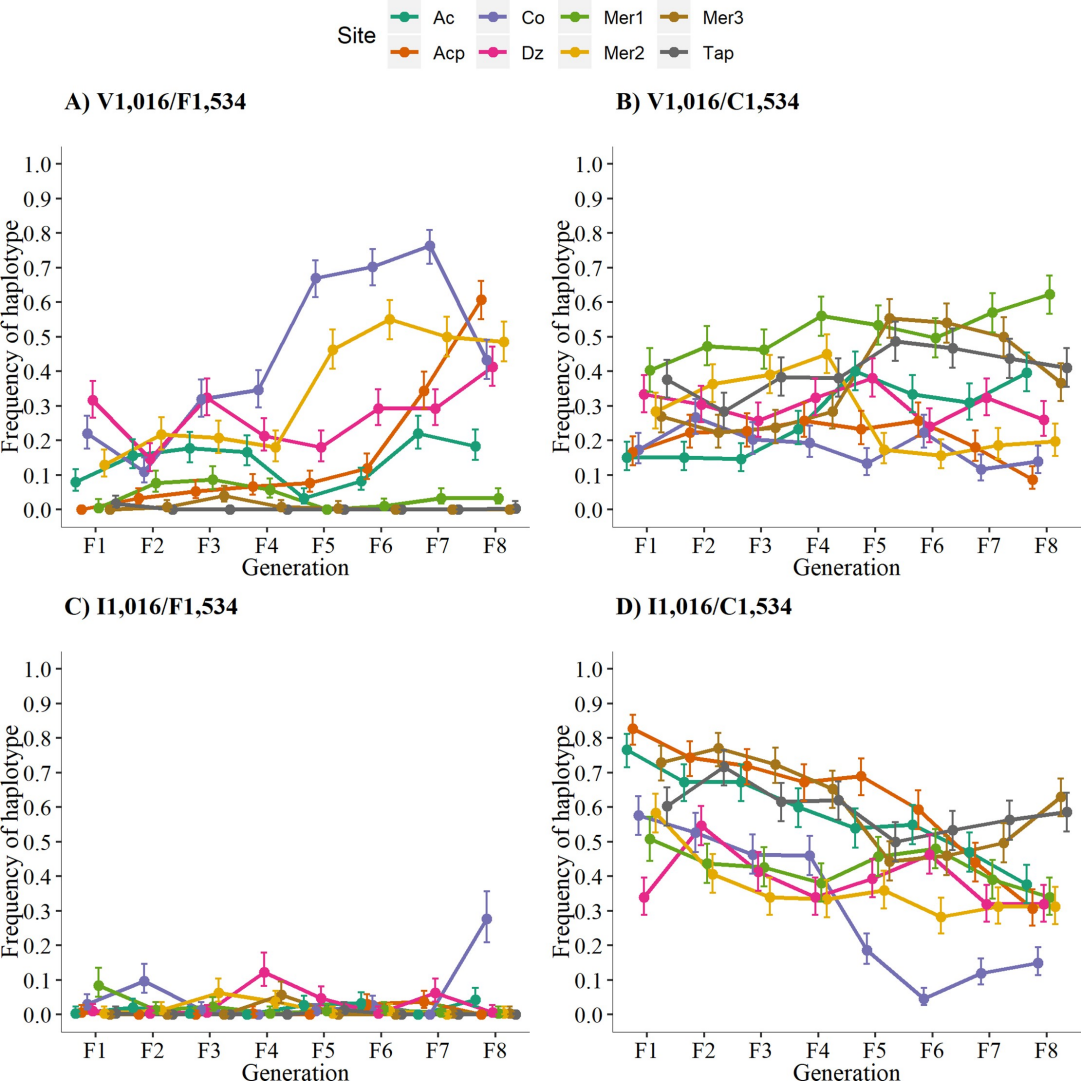
Frequency of the four potential di-locus haplotypes between loci V1,016I and F1,534C over eight generations in the absence of insecticides. Haplotypes: both susceptible alleles in *cis =* V_1,016_/F_1,534_, susceptible V_1,016_ and resistant C_1,534_ alleles in *cis* = V_1,016_/C_1,534_, resistant I_1,016_ and susceptible F_1,534_ alleles in *cis* = I_1,016_/F_1,534_, and resistant I_1,016_ and resistant C_1,534_ alleles in *cis* = I_1,016_/C_1,534_.

The four possible haplotypes resulting from V1,016I and F1,534C are shown in Fig 6. Table 5 displays the correlation and the significance between the haplotype frequencies and generations. The frequency of the susceptible V_1,016_/F_1,534_ haplotype increased over the generations (*r* = 0.323, *p* = 0.009). The frequency of the V_1,016_/C_1,534_ haplotype remained relatively constant (*r* = 0.140, *p* = 0.271). The frequency of the resistant I_1,016_/C_1,534_ haplotype decreased over time (*r* = -0.516, *p* <0.0001) across all collection sites. The frequencies of the I_1,016_/F_1,534_ haplotypes were consistently low (*r* = 0.091, *p* = 0.474) across generations. This same trend has been noted in two previous studies [5] [13], suggesting that low fitness may occur in any mosquito in which I1,016 co-occurs with F1,534.

**Table 5:** Pearson's correlation coefficient and *p* value for four possible haplotypes at loci V1,016I and F1,534C. Haplotypes: both susceptible alleles in *cis =* V_1,016_/F_1,534_, susceptible V_1,016_ and resistant C_1,534_ alleles in *cis* = V_1,016_/C_1,534_, resistant I_1,016_ and susceptible F_1,534_ alleles in *cis* = I_1,016_/F_1,534_, and resistant I_1,016_ and resistant C_1,534_ alleles in *cis* = I_1,016_/C_1,534_.

	V_1,016_/F_1,534_		V_1,016_/C_1,534_		I_1,016_/F_1,534_		I_1,016_/C_1,534_	
Site	Pearson r	*P* value	Pearson r	*P* value	Pearson r	*P* value	Pearson r	*P* value
**Ac**	0.282	0.500	0.868	0.005	0.512	0.195	-0.978	<0.0001
**Acp**	0.842	0.009	-0.356	0.386	0.432	0.285	-0.937	0.001
**Co**	0.754	0.031		-0.566	0.143		0.401	0.325		-0.907	0.002
**Dz**	0.429	0.289	-0.251	0.549	0.130	0.759	-0.388	0.342
**Mer1**	-0.268	0.521	0.873	0.005	-0.672	0.068	-0.618	0.103
**Mer2**	0.882	0.004	-0.633	0.092	-0.339	0.412	-0.770	0.026
**Mer3**	-0.340	0.410	0.677	0.065	-0.031	0.942	-0.688	0.059
**Tap**	-0.480	0.229	0.624	0.099	-0.051	0.904	-0.560	0.149
**Overall**	0.323	0.009	0.140	0.271	0.091	0.474	-0.516	<0.0001

### Temporal analysis of di-locus genotypes

**Fig 7:**
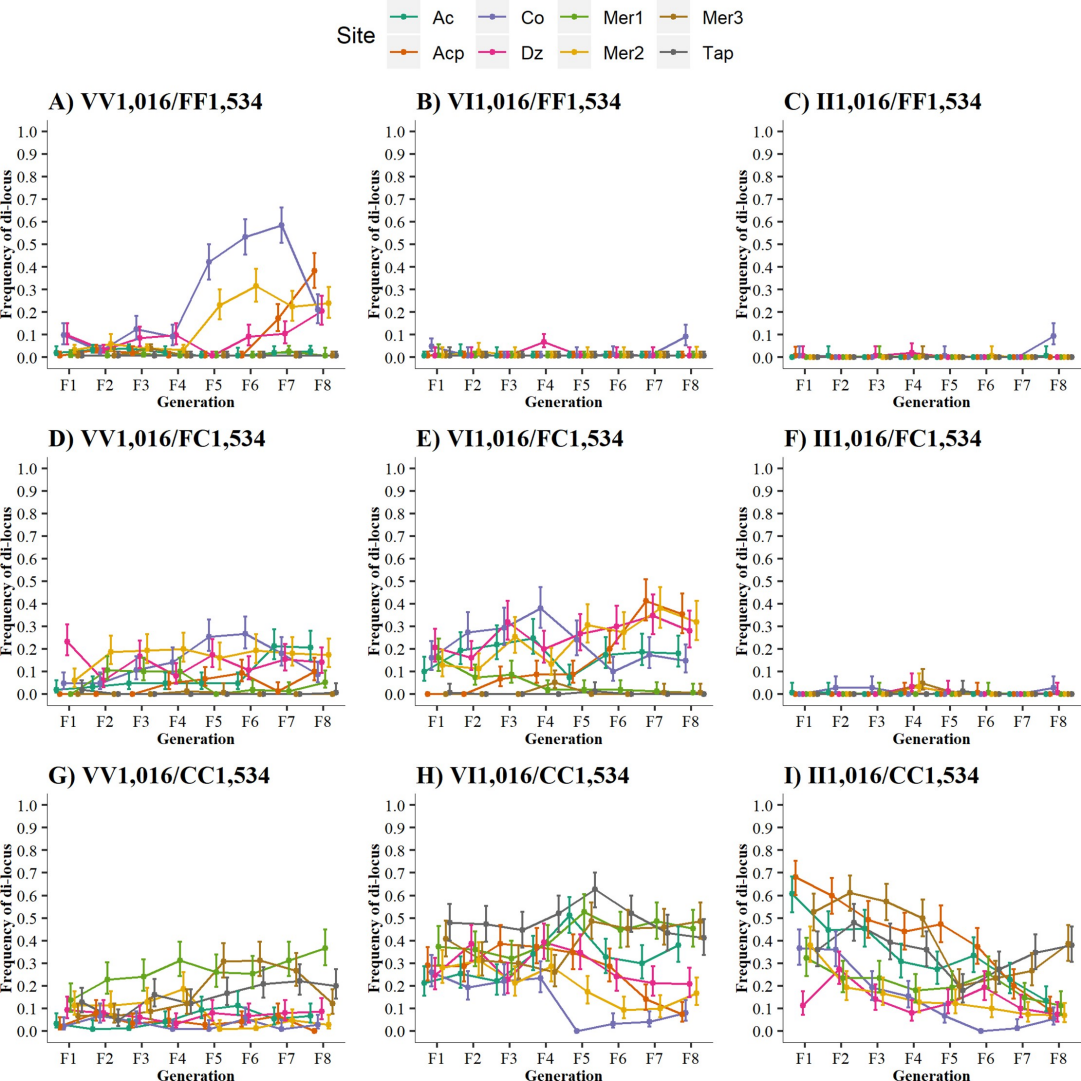
Frequency of the nine di-locus haplotypes over eight generations in the absence of insecticides.

Fig 7 shows the frequency of the nine di-locus genotype combinations (three genotypes at two loci), and Table 6 lists the correlation between frequencies of each di-locus genotype and the generation number without pyrethroid exposure. We estimated the frequencies of the nine genotype combinations in 9,563 mosquitoes. However, the di-locus coefficient among the nine graphs was only significant for the wild type susceptible VV_1,016_/FF_1,534_ and the dual resistant II_1,016_/CC_1,534._ The correlation between generation number and VV_1,016_/FF_1,534_ was positive (*r* = 0.3376, P = 0.0064), indicating an increase in susceptible di-locus genotypes in the absence of insecticides. The correlation of II_1,016_/CC_1,534_ was negative (*r* = -0.5465, P < 0.0001), indicating a decline. The low frequencies of VI_1,016_/FF_1,534_, II_1,016_/FF_1,534_, and II_1,016_/FC_1,534_ in Fig 7B, 7C and 7F are consistent with the hypothesis that low fitness may occur in any mosquito in which I1,016 co-occurs with F1,534. However, Fig 7E identifies an exception to this trend. A substantial proportion of the double heterozygote VI_1016_/FC_1534_ survives to generation F_8._

**Table 6:** Pearson’s correlation coefficient and *p* value for nine dilocus genotypes at loci V1,016I and F1,534.

** **	**VV**_**1,016**_**/FF**_**1,534**_		**VI**_**1,016**_**/FF**_**1,534**_		**II**_**1,016**_**/FF**_**1,534**_	
**Site**	**Pearson r**	***P* value**	**Pearson r**	***P* value**	**Pearson r**	***P* value**
**Ac**	-0.3225	0.4360	-0.4124	0.31	0.1260	0.7663
**Acp**	0.7101	0.0484	-	-	-0.5774	0.1340
**Co**	0.6830	0.0619	0.3135	0.4496	0.5493	0.1585
**Dz**	0.5151	0.1915	-0.0825	0.8461	-0.3693	0.3679
**Mer1**	0.2980	0.4734	-0.5774	0.134	-0.2474	0.5546
**Mer2**	0.8243	0.0118	-0.4124	0.31	0.2474	0.5546
**Mer3**	-0.2531	0.5454	-	-	-0.0825	0.8461
**Tap**	-	-	-	-	-	-
**Across all**	0.3376	0.0064	0.0249	0.8451	0.1370	0.2803
** **	**VV**_**1,016**_**/FC**_**1,534**_		**VI**_**1,016**_**/FC**_**1,534**_		**II**_**1,016**_**/FC**_**1,534**_	
**Site**	**Pearson r**	***P* value**	**Pearson r**	***P* value**	**Pearson r**	***P* value**
**Ac**	0.8132	0.0141	0.1079	0.7993	-0.7559	0.0300
**Acp**	0.7280	0.0406	0.9162	0.0014	0.1260	0.7663
**Co**	0.5219	0.1846	-0.4137	0.3082	-0.0563	0.8946
**Dz**	-0.1429	0.7357	0.6513	0.0802	0.0724	0.8648
**Mer1**	-0.2719	0.5148	-0.8585	0.0064	0.0000	>0.9999
**Mer2**	0.4626	0.2485	0.8381	0.0094	-0.0825	0.8461
**Mer3**	-0.0357	0.9331	0.0494	0.9076	-0.0825	0.8461
**Tap**	-0.3711	0.3654	-0.2143	0.6103	0.0825	0.8461
**Across all**	0.2048	0.1046	0.1817	0.1507	-0.0471	0.712
								
** **	**VV**_**1,016**_**/CC**_**1,534**_		**VI**_**1,016**_**/CC**_**1,534**_		**II**_**1,016**_**/CC**_**1,534**_	
**Site**	**Pearson r**	***P* value**	**Pearson r**	***P* value**	**Pearson r**	***P* value**
**Ac**	0.6439	0.0849	0.5632	0.1461	-0.9390	0.0005
**Acp**	-0.2323	0.5799	-0.6772	0.0651	-0.9628	0.0001
**Co**	-0.2698	0.5182	-0.7866	0.0206	-0.9141	0.0015
**Dz**	0.0398	0.9255	-0.4257	0.293	-0.4131	0.3091
**Mer1**	0.8522	0.0072	0.7112	0.0479	-0.8105	0.0147
**Mer2**	-0.6506	0.0806	-0.8032	0.0164	-0.8622	0.0059
**Mer3**	0.6035	0.1132	0.6312	0.0933	-0.7211	0.0435
**Tap**	0.7993	0.0173	-0.1480	0.7266	-0.3517	0.3930
**Across all**	0.1757	0.165	-0.0899	0.48	-0.5465	<0.0001

### Association between resistance alleles and resistance ratios

**Fig 8:**
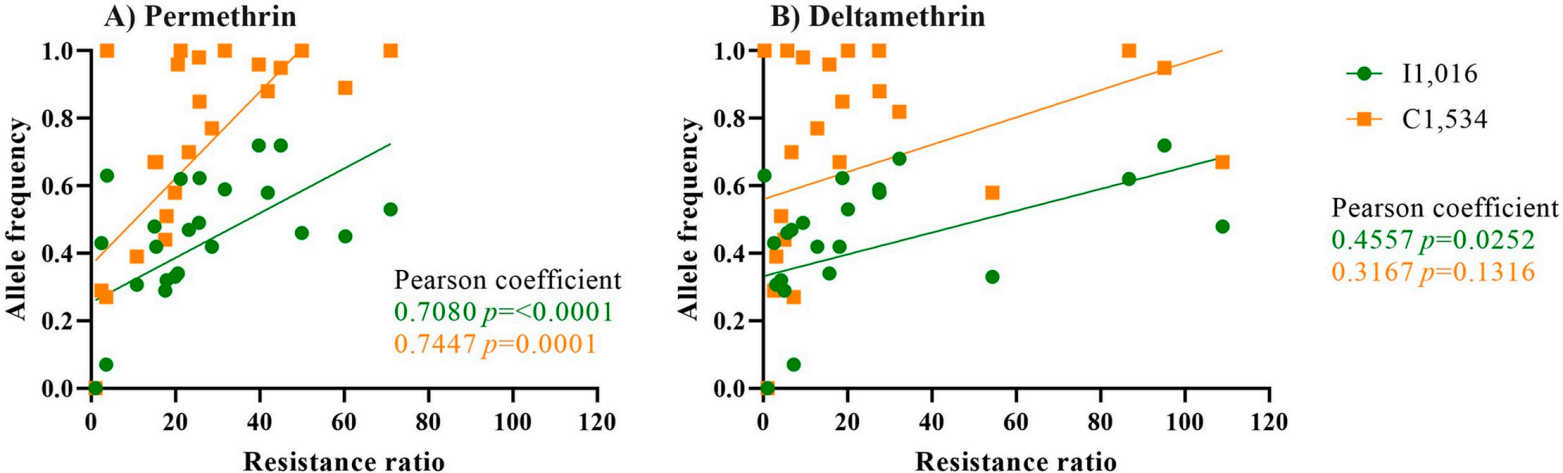
Correlation between the resistant alleles: I1,016 and C1,534 and the pyrethroid resistance ratio calculated for A) permethrin and B) deltamethrin.

Pearson correlation coefficients and their significance were calculated between the frequencies of I1,016 or C1,534 alleles and resistance ratios for permethrin or deltamethrin as determined by bioassay for all eight collections. This analysis was done separately for generations F_3_, F_6_, and F_8._ Table 7 indicates that all correlations were positive in F_3_ and F_8_, but none were significant. All correlations were positive in F_6_, and three were significant. When the results from all three generations were combined, all correlations were positive, and three were significant. Thus, in general, a weak but consistently positive correlation existed between the frequency of the I1,016 or C1,534 alleles and resistance ratios as determined by bioassay (Fig 8).

**Table 7:** Pearson correlation coefficients and their significance between the frequencies of I1,016 or C1,534 alleles and Resistance Ratios (RR) for permethrin or deltamethrin as determined by bioassays for all eight collections.

Generation	Permethrin RR	*P value*	Deltamethrin RR	*P value*
**Locus**	**I1,016**		**I1,016**	
F3 (n = 9)	0.606	0.1489	0.625	0.184
F6 (n = 9)	0.715	0.0303	0.709	0.032
F8 (n = 9)	0.338	0.3732	0.607	0.083
All (n = 27)	0.588	0.002	0.469	0.021
**Locus**	**C1,534**		**C1,534**	
F3 (n = 9)	0.711	0.073	0.66	0.154
F6 (n = 9)	0.822	0.007	0.593	0.093
F8 (n = 9)	0.115	0.768	0.282	0.462
All (n = 27)	0.707	< .0001	0.367	0.078

This correlation also was noted in an earlier study in which I1,016 showed significant protection against permethrin and deltamethrin, whereas F1,534C showed protection against permethrin but not against deltamethrin [14, 15]. The expression of C1,534 in *Xenopus* oocytes and exposure to both pyrethroids demonstrated that the resistant amino acid substitution in C1,534 is sensitive to permethrin but not to deltamethrin, which is consistent with our results.

## Discussion

In this study, we established eight colonies of *Ae*. *aegypti* from the field and maintained them in a pyrethroid-free environment in the laboratory for eight generations. We demonstrated that, in general, the frequency of the *Ae*. *aegypti* pyrethroid-resistance alleles I1,016 and C1,534 decline when released from pyrethroid pressure in the laboratory (Figs 2 and 4, Tables 3 and 5). However, the pattern of decline appeared to be strain dependent, with some strains showing a steady rate of decline (Ac, Acp, and Mer2 in Fig 2; Acp and Mer2 in Fig 4), some showing a shallow decline (Mer1 and Tap in Fig 2; Acp, Mer2, and Co in Fig 4), and others displaying no net change (Dz in Fig 2; Ac, Mer3, and Tap in Fig 4).

A more surprising result was that, in Co and Mer3, the frequencies of I1,016 increased following a precipitous drop. Likewise, the frequencies of C1,534 in Mer1, Mer2, and Co increased after a drop. Such a change might occur if deleterious or lethal recessive mutations are linked to the susceptible allele that became homozygous through continuous inbreeding. However, this theory fails to explain why I1,016 increased simultaneously in all three replicates of Co.

We do not suggest, nor do we know whether the selection pressure in indoor cage studies is the same as or even correlated with outdoor selection pressure. This study only indicates that the loss of pyrethroid resistance is unlikely to follow a smooth linear or exponential decline for any one of a number of reasons. Nor should we expect the decline in resistance to be consistent among collections. Epistatic interactions between alleles may cause nonlinear trends in allele frequencies. Much depends on the genetic background of each population; some populations could take much longer to lose resistance, while others may do so much more rapidly.

A variety of possibilities exist to explain this variance in the gene frequency trajectories among the collections, but this heterogeneity is unlikely to have arisen from small sample sizes. We analyzed three replicates of 50 adult mosquitoes for each of the eight collection sites. Furthermore, 500 adults were used to generate the next generation of eggs for each replicate. The 95% HDI remained narrow in all graphs in Fig 2B and Fig 4B.

Initial conditions may affect the shape of the curve. For example, a curve that begins with initial frequencies close to 1 (Mer3 and Tap in Fig 4A) would begin to decline much later than a curve that begins at 0.6 (Mer1 and Mer2 in Fig 2A). Metabolic resistance may account for much of this heterogeneity. A quantitative trait loci mapping study (QTL) [9] reported that 58.6% of the variation in knockdown could be accounted for by I1,016, but that a number of different QTL located throughout the genome accounted for the remainder of the variation. Saavedra-Rodriguez et al. used the “Aedes Detox” microarray [16] and showed an inverse relationship between I1,016 frequencies and the number of differentially transcribed metabolic genes [17].

Table 5 displays the correlation and the significance between haplotype frequencies and generation. The low frequencies of VI_1,016_/FF_1,534_, II_1,016_/FF_1,534_, and II_1,016_/FC_1,534_ noted in this (Fig 7B, 7C and 7F) and in two previous studies [5] [12] suggest that low fitness may occur in any mosquito in which I1,016 co-occurs with F1,534. This finding, and because C1,534 historically appears before I1,016, suggests that the evolution of the mutations was sequential. If I1,016 had appeared first, it would have co-occurred with F1,534 and would have been eliminated.

Deltamethrin RR appears to be correlated with the I1,016 allele frequency but not with the C1,534 allele frequencies, whereas permethrin RR correlates with both allele frequencies (Table 7). We speculated that other resistance mechanisms are present that could drive resistance to deltamethrin, such as detoxifying enzymes or other mutations in *VGSC* that have not been identified. However, permethrin resistance appears to be driven heavily by both resistance alleles.

The I1,016 amino acid substitution has been found in many resistant populations in the Americas [18–23], and it has been shown that it is in linkage disequilibrium with C1,534 [12]. Recent work has shown that I1,016 is also in very tight disequilibrium with V410L [13].

Interestingly, the I1,016 mutation was functionally expressed in *Xenopus* oocytes and did not show an alteration in the sodium channel sensitivity to either of these pyrethroids [14]. Our data indicate a positive correlation of I1,016 allele frequency with the RR of both pyrethroids. Clearly, a greater understanding is required concerning the role that I1,016 plays in the resistance of *Ae*. *aegypti*. This includes the possibility that V410L [15] may be the residue to which the pyrethroid actually binds.

Since many field populations are resistant to pyrethroids, and few insecticides are available on the market, negative fitness is beneficial for vector control, providing an opportunity for the use of alternative insecticides as pyrethroid resistance is lost. Pyrethroids could then be “saved” to control susceptible populations during a disease outbreak. However, the results in this study suggest that even if dominant wild-type alleles (V1,016 and F1,534) increase in a population in the absence of insecticides, recessive resistance alleles will be hidden and maintained in heterozygotes. Therefore, the initial susceptibility of populations (before the introduction of a pyrethroid) will never be recovered. Furthermore, recent work in Sao Paulo State in Brazil showed that resistance alleles persist in natural populations for at least 11 years [1].

## Supporting information

S1 Table Permethrin and deltamethrin LC50 (ug per bottle) and 95% confidence intervals calculated for eight *Aedes aegypti* colonies from Mexico. Resistance ratios (RR) were calculated at generations F3, F6 and F8 relative to the New Orleans susceptible reference strain.
(DOCX)

S2 Table Di-locus genotype counts for V1,016I and F1,534C by collection site, generation, and replicate number. VV = V1,016 homozygote, VI = V1,016I heterozygote, II = I1,016 homozygote, FF = F1,534 homozygote, FC = F1,534C heterozygote, CC = C1,534 homozygote for *Ae*. *aegypti* in eight generations in absence of pyrethroids.
(DOCX)

S3 Table Frequencies of resistant alleles I1,016 and C1,534 by site and generation in absence of pyrethroids. High-density intervals (HDI) 95% were calculated for each biological replicate separately.
(DOCX)
