# Supplementary material for: Loss of pyrethroid resistance in newly established laboratory colonies of Aedes aegypti
Source: PLoS Negl Trop Dis. 2020 Mar 16;14(3):e0007753. doi: 10.1371/journal.pntd.0007753 (PMC7117762; doi:10.1371/journal.pntd.0007753)
Supplement: S1 Table — Resistance ratios (RR) were calculated at generations F3, F6 and F8 relative to the New Orleans susceptible reference strain. (DOCX) [file pntd.0007753.s001.docx]

| Site | Generation | Insecticide | N | LC50 (ug/bottle) | 95% CI Lower | 95% CI Upper | *p*-value | RR |
| --- | --- | --- | --- | --- | --- | --- | --- | --- |
| NO | F3 | Permethrin | 239 | 0.64 | 0.53 | 0.78 | 0.07 |  |
| NO | F6 | Permethrin | 361 | 0.59 | 0.53 | 0.66 | 0.72 |  |
| NO | F8 | Permethrin | 523 | 0.49 | 0.44 | 0.55 | 0.41 |  |
| Acp | F3 | Permethrin | 818 | 28.78 | 24.34 | 34.05 | 0.24 | 44.9 |
| Acp | F6 | Permethrin | 418 | 15.18 | 12.83 | 17.96 | 0.39 | 25.6 |
| Acp | F8 | Permethrin | 308 | 5.34 | 4.61 | 6.18 | 0.08 | 10.8 |
| Tap | F3 | Permethrin | 497 | 13.58 | 9.96 | 18.53 | 0.87 | 21.2 |
| Tap | F6 | Permethrin | - | - | - | - | - |  |
| Tap | F8 | Permethrin | 545 | 15.61 | 13.94 | 17.76 | 0.87 | 31.7 |
| Mer1 | F3 | Permethrin | 300 | 38.59 | 34.23 | 43.50 | 0.78 | 60.2 |
| Mer1 | F6 | Permethrin | 536 | 15.08 | 11.55 | 19.69 | 0.33 | 25.4 |
| Mer1 | F8 | Permethrin | 307 | 10.11 | 9.49 | 10.76 | 0.61 | 20.5 |
| Mer2 | F3 | Permethrin | - | - | - | - | - |  |
| Mer2 | F6 | Permethrin | 474 | 10.38 | 8.70 | 12.39 | 0.40 | 17.5 |
| Mer2 | F8 | Permethrin | 428 | 8.82 | 7.30 | 10.67 | 0.22 | 17.9 |
| Mer3 | F3 | Permethrin | 534 | 25.45 | 23.03 | 28.11 | 0.55 | 39.7 |
| Mer3 | F6 | Permethrin | 606 | 29.46 | 25.77 | 33.69 | 0.37 | 49.6 |
| Mer3 | F8 | Permethrin | 357 | 1.85 | 1.52 | 2.25 | 0.32 | 3.8 |
| Dz | F3 | Permethrin | 321 | 9.88 | 9.77 | 16.73 | 0.99 | 15.4 |
| Dz | F6 | Permethrin | 375 | 13.68 | 10.42 | 17.98 | 0.15 | 23.0 |
| Dz | F8 | Permethrin | 368 | 9.79 | 7.18 | 13.34 | 0.86 | 19.9 |
| Co | F3 | Permethrin | 385 | 9.63 | 8.08 | 11.49 | 0.95 | 15.0 |
| Co | F6 | Permethrin | 549 | 2.12 | 1.72 | 2.61 | 0.15 | 3.6 |
| Co | F8 | Permethrin | 617 | 1.19 | 1.01 | 1.39 | 0.13 | 2.4 |
| Ac | F3 | Permethrin | - | - | - | - | - |  |
| Ac | F6 | Permethrin | 700 | 24.70 | 21.61 | 28.24 | 0.32 | 41.6 |
| Ac | F8 | Permethrin | 594 | 14.10 | 13.32 | 14.93 | 0.06 | 28.7 |
|  |  |  |  |  |  |  |  |  |
|  |  |  |  |  |  |  |  |  |
|  |  |  |  |  |  |  |  |  |
| NO | F3 | Deltamethrin | 218 | 0.09 | 0.08 | 0.09 | 0.86 |  |
| NO | F6 | Deltamethrin | 336 | 0.12 | 0.10 | 0.14 | 0.27 |  |
| NO | F8 | Deltamethrin | 479 | 0.14 | 0.12 | 0.16 | 0.98 |  |
| Acp | F3 | Deltamethrin | 630 | 8.41 | 7.81 | 9.05 | 0.88 | 95.1 |
| Acp | F6 | Deltamethrin | 316 | 2.27 | 1.86 | 2.76 | 0.53 | 18.8 |
| Acp | F8 | Deltamethrin | 530 | 0.44 | 0.32 | 0.61 | 0.66 | 3.1 |
| Tap | F3 | Deltamethrin | 705 | 7.67 | 6.98 | 8.42 | 0.85 | 86.7 |
| Tap | F6 | Deltamethrin | 819 | 2.43 | 2.25 | 2.62 | 0.10 | 20.1 |
| Tap | F8 | Deltamethrin | 788 | 3.91 | 3.44 | 4.45 | 0.51 | 27.9 |
| Mer1 | F3 | Deltamethrin | - | - | - | - | - |  |
| Mer1 | F6 | Deltamethrin | 567 | 1.14 | 0.95 | 1.36 | 0.62 | 9.4 |
| Mer1 | F8 | Deltamethrin | 289 | 2.23 | 2.11 | 2.36 | 0.10 | 15.9 |
| Mer2 | F3 | Deltamethrin | - | - | - | - | - |  |
| Mer2 | F6 | Deltamethrin | 438 | 0.61 | 0.47 | 0.78 | 0.75 | 5.0 |
| Mer2 | F8 | Deltamethrin | 394 | 0.60 | 0.50 | 0.73 | 0.06 | 4.3 |
| Mer3 | F3 | Deltamethrin | - | - | - | - | - |  |
| Mer3 | F6 | Deltamethrin | 450 | 0.69 | 0.58 | 0.82 | 0.10 | 5.7 |
| Mer3 | F8 | Deltamethrin | 261 | 0.04 | 0.03 | 0.05 | 0.42 | 0.3 |
| Dz | F3 | Deltamethrin | 288 | 1.60 | 0.88 | 2.92 | 0.12 | 18.1 |
| Dz | F6 | Deltamethrin | 675 | 0.81 | 1.22 | 1.43 | 0.46 | 6.7 |
| Dz | F8 | Deltamethrin | - | - | - | - | - |  |
| Co | F3 | Deltamethrin | 385 | 9.63 | 8.08 | 11.49 | 0.95 | 109.0 |
| Co | F6 | Deltamethrin | 378 | 0.87 | 0.78 | 0.97 | 0.35 | 7.2 |
| Co | F8 | Deltamethrin | 269 | 0.37 | 0.28 | 0.48 | 0.79 | 2.6 |
| Ac | F3 | Deltamethrin | 423 | 2.85 | 2.25 | 3.62 | 0.67 | 32.3 |
| Ac | F6 | Deltamethrin | 828 | 3.33 | 2.94 | 3.77 | 0.06 | 27.6 |
| Ac | F8 | Deltamethrin | 304 | 1.82 | 1.34 | 2.47 | 0.24 | 13.0 |
