# Supplementary material for: Loss of pyrethroid resistance in newly established laboratory colonies of Aedes aegypti
Source: PLoS Negl Trop Dis. 2020 Mar 16;14(3):e0007753. doi: 10.1371/journal.pntd.0007753 (PMC7117762; doi:10.1371/journal.pntd.0007753)
Supplement: S2 Table — VV = V1,016 homozygote, VI = V1,016I heterozygote, II = I1,016 homozygote, FF = F1,534 homozygote, FC = F1,534C heterozygote, CC = C1,534 homozygote for Ae. aegypti in eight generations in absence of pyrethroids. (DOCX) [file pntd.0007753.s002.docx]

| **Site** | **Generation** | **Rep** | **N** | **1,016/1,534 di-locus genotype** | | | | | | | | |
| --- | --- | --- | --- | --- | --- | --- | --- | --- | --- | --- | --- | --- |
|  |  |  |  | **VV/FF** | **VV/FC** | **VV/CC** | **IV/FF** | **IV/FC** | **IV/CC** | **II/FF** | **II/FC** | **II/CC** |
| **Acp** |  |  |  |  |  |  |  |  |  |  |  |  |
|  | **F1** | 1 | 50 | 0 | 0 | 0 | 0 | 0 | 12 | 1 | 0 | 37 |
|  |  | 2 | 50 | 0 | 0 | 0 | 0 | 0 | 16 | 0 | 0 | 34 |
|  |  | 3 | 51 | 0 | 0 | 3 | 0 | 0 | 16 | 0 | 0 | 32 |
|  |  | **Total** | **151** | **0** | **0** | **3** | **0** | **0** | **44** | **1** | **0** | **103** |
|  | **F2** | 1 | 50 | 1 | 0 | 4 | 0 | 0 | 13 | 0 | 0 | 32 |
|  |  | 2 | 50 | 3 | 0 | 7 | 0 | 0 | 11 | 0 | 0 | 29 |
|  |  | 3 | 50 | 1 | 0 | 1 | 0 | 0 | 19 | 0 | 0 | 29 |
|  |  | **Total** | **150** | **5** | **0** | **12** | **0** | **0** | **43** | **0** | **0** | **90** |
|  | **F3** | 1 | 50 | 0 | 0 | 2 | 0 | 0 | 18 | 0 | 0 | 30 |
|  |  | 2 | 50 | 1 | 0 | 2 | 0 | 2 | 21 | 0 | 0 | 24 |
|  |  | 3 | 50 | 2 | 0 | 1 | 0 | 8 | 19 | 0 | 0 | 20 |
|  |  | **Total** | **150** | **3** | **0** | **5** | **0** | **10** | **58** | **0** | **0** | **74** |
|  | **F4** | 1 | 50 | 0 | 2 | 4 | 0 | 2 | 20 | 0 | 0 | 22 |
|  |  | 2 | 50 | 0 | 2 | 0 | 0 | 7 | 18 | 0 | 1 | 22 |
|  |  | 3 | 50 | 0 | 3 | 3 | 0 | 4 | 18 | 0 | 0 | 22 |
|  |  | **Total** | **150** | **0** | **7** | **7** | **0** | **13** | **56** | **0** | **1** | **66** |
|  | **F5** | 1 | 50 | 0 | 3 | 3 | 0 | 5 | 15 | 0 | 0 | 24 |
|  |  | 2 | 50 | 0 | 3 | 1 | 0 | 4 | 20 | 0 | 0 | 22 |
|  |  | 3 | 50 | 0 | 4 | 0 | 0 | 4 | 17 | 0 | 0 | 25 |
|  |  | **Total** | **150** | **0** | **10** | **4** | **0** | **13** | **52** | **0** | **0** | **71** |
|  | **F6** | 1 | 50 | 0 | 8 | 0 | 0 | 12 | 9 | 0 | 1 | 20 |
|  |  | 2 | 50 | 0 | 3 | 1 | 0 | 10 | 13 | 0 | 0 | 23 |
|  |  | 3 | 50 | 0 | 3 | 5 | 0 | 8 | 21 | 0 | 0 | 13 |
|  |  | **Total** | **150** | **0** | **14** | **6** | **0** | **30** | **43** | **0** | **1** | **56** |
|  | **F7** | 1 | 50 | 11 | 0 | 0 | 0 | 29 | 0 | 0 | 0 | 10 |
|  |  | 2 | 50 | 2 | 1 | 9 | 0 | 11 | 18 | 0 | 0 | 9 |
|  |  | 3 | 50 | 12 | 1 | 1 | 0 | 22 | 3 | 0 | 0 | 11 |
|  |  | **Total** | **150** | **25** | **2** | **10** | **0** | **62** | **21** | **0** | **0** | **30** |
|  | **F8** | 1 | 50 | 22 | 3 | 0 | 0 | 13 | 5 | 0 | 0 | 7 |
|  |  | 2 | 50 | 14 | 7 | 0 | 0 | 20 | 6 | 0 | 0 | 3 |
|  |  | 3 | 50 | 21 | 5 | 0 | 0 | 20 | 0 | 0 | 0 | 4 |
|  |  | **Total** | **150** | **57** | **15** | **0** | **0** | **53** | **11** | **0** | **0** | **14** |
| **Tap** |  |  |  |  |  |  |  |  |  |  |  |  |
|  | **F1** | 1 | 50 | 0 | 0 | 7 | 0 | 0 | 25 | 0 | 0 | 18 |
|  |  | 2 | 50 | 0 | 0 | 9 | 0 | 0 | 26 | 0 | 0 | 15 |
|  |  | 3 | 50 | 0 | 3 | 3 | 1 | 1 | 21 | 0 | 0 | 21 |
|  |  | Total | 150 | 0 | 3 | 19 | 1 | 1 | 72 | 0 | 0 | 54 |
|  | **F2** | 1 | **50** | **0** | **0** | **2** | **0** | **0** | **19** | **0** | **0** | **29** |
|  |  | 2 | 50 | 0 | 0 | 4 | 0 | 0 | 21 | 0 | 0 | 25 |
|  |  | 3 | 50 | 0 | 0 | 1 | 0 | 0 | 31 | 0 | 0 | 18 |
|  |  | **Total** | **150** | **0** | **0** | **7** | **0** | **0** | **71** | **0** | **0** | **72** |
|  | **F3** | 1 | **50** | **0** | **0** | **8** | **0** | **0** | **24** | **0** | **0** | **18** |
|  |  | 2 | 50 | 0 | 0 | 8 | 0 | 0 | 20 | 0 | 0 | 22 |
|  |  | 3 | 50 | 0 | 0 | 8 | 0 | 0 | 23 | 0 | 0 | 19 |
|  |  | **Total** | **150** | **0** | **0** | **24** | **0** | **0** | **67** | **0** | **0** | **59** |
|  | **F4** | 1 | **50** | **0** | **0** | **7** | **0** | **0** | **24** | **0** | **0** | **19** |
|  |  | 2 | 50 | 0 | 0 | 5 | 0 | 0 | 30 | 0 | 0 | 15 |
|  |  | 3 | 50 | 0 | 0 | 6 | 0 | 0 | 24 | 0 | 0 | 20 |
|  |  | **Total** | **150** | **0** | **0** | **18** | **0** | **0** | **78** | **0** | **0** | **54** |

Table 1. (Continued)

| **Site** | **Generation** | **Rep** | **N** | **1,534/1,016 di-locus genotype** | | | | | | | | |
| --- | --- | --- | --- | --- | --- | --- | --- | --- | --- | --- | --- | --- |
|  |  |  |  | **VV/FF** | **VV/FC** | **VV/CC** | **IV/FF** | **IV/FC** | **IV/CC** | **II/FF** | **II/FC** | **II/CC** |
|  | **F5** | 1 | **50** | **0** | **0** | **12** | **0** | **1** | **30** | **0** | **0** | **7** |
|  |  | 2 | 50 | 0 | 0 | 4 | 0 | 0 | 30 | 0 | 2 | 14 |
|  |  | 3 | 50 | 0 | 0 | 9 | 0 | 1 | 34 | 0 | 0 | 6 |
|  |  | **Total** | **150** | **0** | **0** | **25** | **0** | **2** | **94** | **0** | **2** | **27** |
|  | **F6** | 1 | **50** | **0** | **0** | **9** | **0** | **0** | **29** | **0** | **0** | **12** |
|  |  | 2 | 50 | 0 | 0 | 15 | 0 | 0 | 22 | 0 | 0 | 13 |
|  |  | 3 | 50 | 0 | 0 | 7 | 0 | 0 | 27 | 0 | 0 | 16 |
|  |  | **Total** | **150** | **0** | **0** | **31** | **0** | **0** | **78** | **0** | **0** | **41** |
|  | **F7** | 1 | **50** | **0** | **0** | **13** | **0** | **0** | **22** | **0** | **0** | **15** |
|  |  | 2 | 50 | 0 | 0 | 7 | 0 | 0 | 24 | 0 | 0 | 19 |
|  |  | 3 | 50 | 0 | 0 | 13 | 0 | 0 | 19 | 0 | 0 | 18 |
|  |  | **Total** | **150** | **0** | **0** | **33** | **0** | **0** | **65** | **0** | **0** | **52** |
|  | **F8** | 1 | **50** | **0** | **0** | **13** | **0** | **0** | **15** | **0** | **0** | **22** |
|  |  | 2 | 50 | 0 | 1 | 9 | 0 | 0 | 23 | 0 | 0 | 17 |
|  |  | 3 | 50 | 0 | 0 | 8 | 0 | 0 | 24 | 0 | 0 | 18 |
|  |  | **Total** | **150** | **0** | **1** | **30** | **0** | **0** | **62** | **0** | **0** | **57** |
| **Mer1** |  |  |  |  |  |  |  |  |  |  |  |  |
|  | **F1** | 1 | 50 | 0 | 0 | 8 | 1 | 10 | 14 | 0 | 0 | 17 |
|  |  | 2 | 50 | 0 | 0 | 6 | 0 | 6 | 24 | 0 | 0 | 14 |
|  |  | 3 | 18 | 0 | 0 | 2 | 0 | 3 | 6 | 0 | 0 | 7 |
|  |  | **Total** | **118** | **0** | **0** | **16** | **1** | **19** | **44** | **0** | **0** | **38** |
|  | **F2** | 1 | 50 | 0 | 7 | 13 | 0 | 1 | 16 | 0 | 0 | 13 |
|  |  | 2 | 50 | 0 | 6 | 9 | 0 | 6 | 20 | 0 | 0 | 9 |
|  |  | 3 | 50 | 0 | 3 | 12 | 0 | 4 | 18 | 0 | 0 | 13 |
|  |  | **Total** | **150** | **0** | **16** | **34** | **0** | **11** | **54** | **0** | **0** | **35** |
|  | **F3** | 1 | 50 | 0 | 13 | 6 | 0 | 9 | 6 | 0 | 1 | 15 |
|  |  | 2 | 50 | 1 | 0 | 16 | 0 | 1 | 22 | 1 | 0 | 9 |
|  |  | 3 | 50 | 0 | 2 | 14 | 0 | 3 | 20 | 0 | 0 | 11 |
|  |  | **Total** | **150** | **1** | **15** | **36** | **0** | **13** | **48** | **1** | **1** | **35** |
|  | **F4** | 1 | 50 | 0 | 5 | 21 | 0 | 1 | 17 | 0 | 0 | 6 |
|  |  | 2 | 50 | 0 | 5 | 12 | 0 | 1 | 18 | 0 | 0 | 14 |
|  |  | 3 | 50 | 0 | 5 | 14 | 0 | 1 | 23 | 0 | 0 | 7 |
|  |  | **Total** | **150** | **0** | **15** | **47** | **0** | **3** | **58** | **0** | **0** | **27** |
|  | **F5** | 1 | 50 | 0 | 0 | 13 | 0 | 0 | 29 | 0 | 0 | 8 |
|  |  | 2 | 50 | 0 | 0 | 16 | 0 | 0 | 25 | 0 | 0 | 9 |
|  |  | 3 | 50 | 0 | 0 | 10 | 0 | 3 | 25 | 0 | 0 | 12 |
|  |  | **Total** | **150** | **0** | **0** | **39** | **0** | **3** | **79** | **0** | **0** | **29** |
|  | **F6** | 1 | 50 | 0 | 1 | 10 | 0 | 0 | 29 | 0 | 0 | 10 |
|  |  | 2 | 50 | 0 | 1 | 14 | 0 | 2 | 22 | 0 | 1 | 10 |
|  |  | 3 | 50 | 0 | 1 | 14 | 0 | 1 | 16 | 0 | 0 | 18 |
|  |  | **Total** | **150** | **0** | **3** | **38** | **0** | **3** | **67** | **0** | **1** | **38** |
|  | **F7** | 1 | 50 | 1 | 1 | 10 | 0 | 2 | 28 | 0 | 0 | 8 |
|  |  | 2 | 50 | 3 | 0 | 19 | 0 | 0 | 24 | 0 | 0 | 4 |
|  |  | 3 | 50 | 0 | 1 | 18 | 0 | 0 | 21 | 0 | 0 | 10 |
|  |  | **Total** | **150** | **4** | **2** | **47** | **0** | **2** | **73** | **0** | **0** | **22** |
|  | **F8** | 1 | 50 | 1 | 3 | 27 | 0 | 1 | 14 | 0 | 0 | 4 |
|  |  | 2 | 50 | 0 | 2 | 13 | 0 | 0 | 25 | 0 | 0 | 10 |
|  |  | 3 | 50 | 0 | 3 | 15 | 0 | 0 | 29 | 0 | 0 | 3 |
|  |  | **Total** | **150** | **1** | **8** | **55** | **0** | **1** | **68** | **0** | **0** | **17** |
| **Mer2** |  |  |  |  |  |  |  |  |  |  |  |  |
|  | **F1** | 1 | 50 | 2 | 4 | 7 | 1 | 6 | 13 | 0 | 0 | 17 |
|  |  | 2 | 50 | 2 | 2 | 7 | 0 | 9 | 11 | 0 | 0 | 19 |
|  |  | 3 | 50 | 1 | 3 | 3 | 0 | 4 | 18 | 0 | 0 | 21 |

Continued

Table 1. (Continued)

| **Site** | **Generation** | **Rep** | **N** | **1,534/1,016 di-locus genotype** | | | | | | | | |
| --- | --- | --- | --- | --- | --- | --- | --- | --- | --- | --- | --- | --- |
|  |  |  |  | **VV/FF** | **VV/FC** | **VV/CC** | **IV/FF** | **IV/FC** | **IV/CC** | **II/FF** | **II/FC** | **II/CC** |
|  |  | **Total** | **150** | **5** | **9** | **17** | **1** | **19** | **42** | **0** | **0** | **57** |
|  | **F2** | 1 | 50 | 2 | 11 | 7 | 0 | 4 | 10 | 0 | 0 | 16 |
|  |  | 2 | 50 | 2 | 8 | 4 | 0 | 8 | 19 | 0 | 0 | 9 |
|  |  | 3 | 50 | 4 | 9 | 6 | 4 | 5 | 18 | 0 | 0 | 4 |
|  |  | Total | 150 | 8 | 28 | 17 | 4 | 17 | 47 | 0 | 0 | 29 |
|  | **F3** | 1 | 50 | 1 | 10 | 7 | 1 | 8 | 12 | 0 | 0 | 11 |
|  |  | 2 | 50 | 1 | 12 | 4 | 0 | 18 | 12 | 0 | 0 | 3 |
|  |  | 3 | 50 | 4 | 7 | 8 | 0 | 12 | 8 | 0 | 0 | 11 |
|  |  | Total | 150 | 6 | 29 | 19 | 1 | 38 | 32 | 0 | 0 | 25 |
|  | **F4** | 1 | 50 | 2 | 9 | 6 | 0 | 8 | 20 | 0 | 0 | 5 |
|  |  | 2 | 50 | 2 | 12 | 8 | 0 | 6 | 10 | 0 | 1 | 11 |
|  |  | 3 | 50 | 1 | 9 | 14 | 0 | 6 | 13 | 0 | 4 | 3 |
|  |  | Total | 150 | 5 | 30 | 28 | 0 | 20 | 43 | 0 | 5 | 19 |
|  | **F5** | 1 | 50 | 8 | 11 | 0 | 0 | 15 | 9 | 0 | 0 | 7 |
|  |  | 2 | 50 | 11 | 10 | 0 | 0 | 12 | 9 | 0 | 0 | 8 |
|  |  | 3 | 50 | 15 | 3 | 1 | 1 | 19 | 8 | 0 | 0 | 3 |
|  |  | Total | 150 | 34 | 24 | 1 | 1 | 46 | 26 | 0 | 0 | 18 |
|  | **F6** | 1 | 50 | 22 | 9 | 0 | 1 | 12 | 4 | 0 | 0 | 2 |
|  |  | 2 | 50 | 13 | 8 | 0 | 0 | 14 | 7 | 0 | 0 | 8 |
|  |  | 3 | 50 | 12 | 12 | 2 | 0 | 15 | 3 | 1 | 0 | 5 |
|  |  | Total | 150 | 47 | 29 | 2 | 1 | 41 | 14 | 1 | 0 | 15 |
|  | **F7** | 1 | 50 | 12 | 9 | 0 | 0 | 22 | 5 | 0 | 0 | 2 |
|  |  | 2 | 50 | 14 | 6 | 5 | 0 | 17 | 5 | 0 | 0 | 3 |
|  |  | 3 | 50 | 7 | 12 | 2 | 0 | 18 | 5 | 0 | 0 | 6 |
|  |  | Total | 150 | 33 | 27 | 7 | 0 | 57 | 15 | 0 | 0 | 11 |
|  | **F8** | 1 | 50 | 11 | 15 | 2 | 0 | 11 | 8 | 0 | 0 | 3 |
|  |  | 2 | 50 | 14 | 4 | 0 | 0 | 19 | 8 | 0 | 0 | 5 |
|  |  | 3 | 44 | 9 | 6 | 2 | 1 | 16 | 8 | 0 | 0 | 2 |
|  |  | Total | 144 | 34 | 25 | 4 | 1 | 46 | 24 | 0 | 0 | 10 |
| **Mer3** |  |  |  |  |  |  |  |  |  |  |  |  |
|  | **F1** | 1 | 50 | 0 | 0 | 7 | 0 | 0 | 23 | 0 | 0 | 20 |
|  |  | 2 | 50 | 0 | 0 | 2 | 0 | 0 | 15 | 0 | 0 | 33 |
|  |  | 3 | 50 | 0 | 0 | 1 | 0 | 0 | 23 | 0 | 0 | 26 |
|  |  | Total | 150 | 0 | 0 | 10 | 0 | 0 | 61 | 0 | 0 | 79 |
|  | **F2** | 1 | 50 | 0 | 0 | 2 | 0 | 0 | 15 | 0 | 0 | 33 |
|  |  | 2 | 50 | 1 | 0 | 5 | 0 | 0 | 16 | 0 | 0 | 28 |
|  |  | 3 | 50 | 0 | 0 | 3 | 0 | 0 | 16 | 0 | 0 | 31 |
|  |  | Total | 150 | 1 | 0 | 10 | 0 | 0 | 47 | 0 | 0 | 92 |
|  | **F3** | 1 | 50 | 5 | 0 | 5 | 0 | 0 | 17 | 0 | 0 | 23 |
|  |  | 2 | 50 | 1 | 0 | 6 | 0 | 0 | 12 | 0 | 0 | 31 |
|  |  | 3 | 50 | 0 | 0 | 2 | 0 | 0 | 16 | 0 | 0 | 32 |
|  |  | Total | 150 | 6 | 0 | 13 | 0 | 0 | 45 | 0 | 0 | 86 |
|  | **F4** | 1 | 50 | 0 | 0 | 2 | 0 | 0 | 13 | 0 | 0 | 35 |
|  |  | 2 | 50 | 0 | 1 | 9 | 0 | 0 | 17 | 0 | 0 | 23 |
|  |  | 3 | 50 | 0 | 1 | 7 | 0 | 8 | 9 | 1 | 7 | 17 |
|  |  | Total | 150 | 0 | 2 | 18 | 0 | 8 | 39 | 1 | 7 | 75 |
|  | **F5** | 1 | 50 | 0 | 1 | 17 | 0 | 0 | 23 | 0 | 0 | 9 |
|  |  | 2 | 50 | 0 | 0 | 11 | 0 | 0 | 26 | 0 | 0 | 13 |
|  |  | 3 | 50 | 0 | 0 | 18 | 0 | 0 | 24 | 0 | 0 | 8 |
|  |  | Total | 150 | 0 | 1 | 46 | 0 | 0 | 73 | 0 | 0 | 30 |
|  | **F6** | 1 | 50 | 0 | 0 | 17 | 0 | 0 | 22 | 0 | 0 | 11 |
|  |  | 2 | 50 | 0 | 0 | 16 | 0 | 0 | 20 | 0 | 0 | 14 |
|  |  | 3 | 50 | 0 | 0 | 14 | 0 | 0 | 26 | 0 | 0 | 10 |

Continued

Table 1. (Continued)

| **Site** | **Generation** | **Rep** | **N** | **1,534/1,016 di-locus genotype** | | | | | | | | |
| --- | --- | --- | --- | --- | --- | --- | --- | --- | --- | --- | --- | --- |
|  |  |  |  | **VV/FF** | **VV/FC** | **VV/CC** | **IV/FF** | **IV/FC** | **IV/CC** | **II/FF** | **II/FC** | **II/CC** |
|  |  | Total | 150 | 0 | 0 | 47 | 0 | 0 | 68 | 0 | 0 | 35 |
|  | **F7** | 1 | 50 | 0 | 0 | 17 | 0 | 0 | 28 | 0 | 0 | 5 |
|  |  | 2 | 50 | 0 | 0 | 15 | 0 | 1 | 22 | 0 | 0 | 12 |
|  |  | 3 | 50 | 0 | 0 | 8 | 0 | 0 | 19 | 0 | 0 | 23 |
|  |  | Total | 150 | 0 | 0 | 40 | 0 | 1 | 69 | 0 | 0 | 40 |
|  | **F8** | 1 | 50 | 0 | 0 | 4 | 0 | 0 | 29 | 0 | 0 | 17 |
|  |  | 2 | 50 | 0 | 0 | 10 | 0 | 1 | 16 | 0 | 0 | 23 |
|  |  | 3 | 50 | 0 | 0 | 4 | 0 | 0 | 28 | 0 | 0 | 18 |
|  |  | Total | 150 | 0 | 0 | 18 | 0 | 1 | 73 | 0 | 0 | 58 |
| **Dz** |  |  |  |  |  |  |  |  |  |  |  |  |
|  | **F1** | 1 | 50 | 4 | 14 | 3 | 1 | 5 | 15 | 0 | 0 | 8 |
|  |  | 2 | 50 | 7 | 12 | 7 | 0 | 10 | 9 | 1 | 0 | 4 |
|  |  | 3 | 50 | 3 | 9 | 4 | 0 | 16 | 13 | 0 | 0 | 5 |
|  |  | Total | 150 | 14 | 35 | 14 | 1 | 31 | 37 | 1 | 0 | 17 |
|  | **F2** | 1 | 50 | 1 | 0 | 2 | 0 | 0 | 24 | 0 | 0 | 23 |
|  |  | 2 | 50 | 1 | 1 | 4 | 0 | 11 | 19 | 0 | 0 | 14 |
|  |  | 3 | 50 | 3 | 8 | 6 | 1 | 13 | 15 | 0 | 0 | 4 |
|  |  | Total | 150 | 5 | 9 | 12 | 1 | 24 | 58 | 0 | 0 | 41 |
|  | **F3** | 1 | 50 | 5 | 5 | 3 | 0 | 16 | 9 | 1 | 0 | 11 |
|  |  | 2 | 50 | 6 | 9 | 2 | 0 | 17 | 12 | 0 | 0 | 4 |
|  |  | 3 | 50 | 1 | 11 | 4 | 0 | 15 | 13 | 0 | 0 | 6 |
|  |  | Total | 150 | 12 | 25 | 9 | 0 | 48 | 34 | 1 | 0 | 21 |
|  | **F4** | 1 | 50 | 8 | 5 | 0 | 7 | 7 | 20 | 3 | 0 | 0 |
|  |  | 2 | 50 | 5 | 5 | 3 | 0 | 14 | 19 | 0 | 1 | 3 |
|  |  | 3 | 50 | 1 | 2 | 2 | 3 | 9 | 20 | 0 | 4 | 9 |
|  |  | Total | 150 | 14 | 12 | 5 | 10 | 30 | 59 | 3 | 5 | 12 |
|  | **F5** | 1 | 50 | 0 | 10 | 3 | 0 | 11 | 21 | 0 | 0 | 5 |
|  |  | 2 | 50 | 0 | 7 | 4 | 0 | 12 | 22 | 0 | 2 | 3 |
|  |  | 3 | 50 | 0 | 9 | 5 | 0 | 17 | 9 | 0 | 0 | 10 |
|  |  | Total | 150 | 0 | 26 | 12 | 0 | 40 | 52 | 0 | 2 | 18 |
|  | **F6** | 1 | 50 | 3 | 2 | 6 | 0 | 18 | 13 | 0 | 0 | 8 |
|  |  | 2 | 50 | 6 | 5 | 2 | 0 | 15 | 11 | 0 | 0 | 11 |
|  |  | 3 | 50 | 4 | 9 | 2 | 1 | 12 | 12 | 0 | 0 | 10 |
|  |  | Total | 150 | 13 | 16 | 10 | 1 | 45 | 36 | 0 | 0 | 29 |
|  | **F7** | 1 | 50 | 5 | 4 | 8 | 0 | 18 | 12 | 0 | 0 | 3 |
|  |  | 2 | 50 | 6 | 11 | 1 | 0 | 17 | 10 | 0 | 0 | 5 |
|  |  | 3 | 50 | 4 | 8 | 3 | 1 | 17 | 10 | 0 | 0 | 7 |
|  |  | Total | 150 | 15 | 23 | 12 | 1 | 52 | 32 | 0 | 0 | 15 |
|  | **F8** | 1 | 50 | 16 | 7 | 0 | 0 | 19 | 5 | 0 | 0 | 3 |
|  |  | 2 | 50 | 9 | 10 | 4 | 0 | 14 | 11 | 0 | 0 | 2 |
|  |  | 3 | 50 | 5 | 4 | 9 | 1 | 9 | 15 | 0 | 1 | 6 |
|  |  | Total | 150 | 30 | 21 | 13 | 1 | 42 | 31 | 0 | 1 | 11 |
| **Co** |  |  |  |  |  |  |  |  |  |  |  |  |
|  | **F1** | 1 | 50 | 2 | 2 | 0 | 1 | 4 | 18 | 0 | 0 | 23 |
|  |  | 2 | 50 | 10 | 2 | 1 | 5 | 11 | 10 | 0 | 0 | 11 |
|  |  | 3 | 50 | 2 | 3 | 2 | 1 | 9 | 11 | 1 | 0 | 21 |
|  |  | Total | 150 | 14 | 7 | 3 | 7 | 24 | 39 | 1 | 0 | 55 |
|  | **F2** | 1 | 50 | 0 | 1 | 1 | 1 | 1 | 10 | 0 | 4 | 32 |
|  |  | 2 | 50 | 2 | 3 | 3 | 0 | 17 | 10 | 0 | 0 | 15 |
|  |  | 3 | 50 | 2 | 3 | 6 | 0 | 23 | 9 | 0 | 0 | 7 |
|  |  | Total | 150 | 4 | 7 | 10 | 1 | 41 | 29 | 0 | 4 | 54 |
|  | **F3** | 1 | 50 | 7 | 5 | 1 | 0 | 15 | 8 | 0 | 3 | 11 |
|  |  | 2 | 50 | 4 | 7 | 1 | 0 | 16 | 16 | 0 | 1 | 5 |

Continued

Table 1. (Continued)

| **Site** | **Generation** | **Rep** | **N** | **1,534/1,016 di-locus genotype** | | | | | | | | |
| --- | --- | --- | --- | --- | --- | --- | --- | --- | --- | --- | --- | --- |
|  |  |  |  | **VV/FF** | **VV/FC** | **VV/CC** | **IV/FF** | **IV/FC** | **IV/CC** | **II/FF** | **II/FC** | **II/CC** |
|  |  | 3 | 50 | 7 | 4 | 4 | 0 | 13 | 9 | 0 | 0 | 13 |
|  |  | Total | 150 | 18 | 16 | 6 | 0 | 44 | 33 | 0 | 4 | 29 |
|  | **F4** | 1 | 50 | 1 | 10 | 0 | 0 | 16 | 16 | 0 | 0 | 7 |
|  |  | 2 | 50 | 2 | 7 | 0 | 0 | 24 | 13 | 0 | 0 | 4 |
|  |  | 3 | 50 | 10 | 4 | 1 | 0 | 17 | 6 | 0 | 0 | 12 |
|  |  | Total | 150 | 13 | 21 | 1 | 0 | 57 | 35 | 0 | 0 | 23 |
|  | **F5** | 1 | 50 | 22 | 13 | 0 | 1 | 11 | 0 | 1 | 0 | 2 |
|  |  | 2 | 50 | 24 | 12 | 1 | 0 | 10 | 0 | 0 | 0 | 3 |
|  |  | 3 | 50 | 17 | 13 | 0 | 0 | 15 | 0 | 0 | 0 | 5 |
|  |  | Total | 150 | 63 | 38 | 1 | 1 | 36 | 0 | 1 | 0 | 10 |
|  | **F6** | 1 | 50 | 29 | 11 | 4 | 0 | 4 | 2 | 0 | 0 | 0 |
|  |  | 2 | 50 | 23 | 18 | 2 | 1 | 6 | 0 | 0 | 0 | 0 |
|  |  | 3 | 50 | 28 | 11 | 2 | 1 | 5 | 3 | 0 | 0 | 0 |
|  |  | Total | 150 | 80 | 40 | 8 | 2 | 15 | 5 | 0 | 0 | 0 |
|  | **F7** | 1 | 50 | 16 | 16 | 1 | 0 | 11 | 5 | 0 | 0 | 1 |
|  |  | 2 | 50 | 34 | 7 | 0 | 0 | 7 | 1 | 0 | 0 | 1 |
|  |  | 3 | 50 | 38 | 4 | 0 | 0 | 8 | 0 | 0 | 0 | 0 |
|  |  | Total | 150 | 88 | 27 | 1 | 0 | 26 | 6 | 0 | 0 | 2 |
|  | **F8** | 1 | 50 | 4 | 3 | 0 | 21 | 3 | 2 | 12 | 4 | 1 |
|  |  | 2 | 50 | 18 | 3 | 0 | 21 | 6 | 0 | 2 | 0 | 0 |
|  |  | 3 | 50 | 9 | 7 | 4 | 0 | 13 | 10 | 0 | 0 | 7 |
|  |  | Total | 150 | 31 | 13 | 4 | 42 | 22 | 12 | 14 | 4 | 8 |
| **Ac** |  |  |  |  |  |  |  |  |  |  |  |  |
|  | **F1** | 1 | 50 | 0 | 2 | 2 | 0 | 4 | 9 | 0 | 0 | 33 |
|  |  | 2 | 50 | 1 | 1 | 1 | 0 | 5 | 11 | 0 | 0 | 31 |
|  |  | 3 | 50 | 2 | 0 | 2 | 0 | 6 | 12 | 0 | 1 | 27 |
|  |  | Total | 150 | 3 | 3 | 5 | 0 | 15 | 32 | 0 | 1 | 91 |
|  | **F2** | 1 | 50 | 5 | 3 | 0 | 2 | 17 | 11 | 0 | 0 | 12 |
|  |  | 2 | 50 | 0 | 1 | 0 | 1 | 7 | 16 | 0 | 0 | 25 |
|  |  | 3 | 50 | 0 | 1 | 1 | 0 | 5 | 11 | 1 | 1 | 30 |
|  |  | Total | 150 | 5 | 5 | 1 | 3 | 29 | 38 | 1 | 1 | 67 |
|  | **F3** | 1 | 50 | 5 | 3 | 2 | 1 | 14 | 10 | 0 | 0 | 15 |
|  |  | 2 | 50 | 0 | 3 | 0 | 0 | 12 | 8 | 0 | 0 | 27 |
|  |  | 3 | 50 | 1 | 1 | 0 | 0 | 7 | 15 | 0 | 0 | 26 |
|  |  | Total | 150 | 6 | 7 | 2 | 1 | 33 | 33 | 0 | 0 | 68 |
|  | **F4** | 1 | 50 | 0 | 3 | 2 | 0 | 16 | 14 | 0 | 0 | 15 |
|  |  | 2 | 50 | 0 | 2 | 2 | 0 | 12 | 16 | 0 | 0 | 18 |
|  |  | 3 | 50 | 3 | 2 | 2 | 0 | 9 | 21 | 0 | 0 | 13 |
|  |  | Total | 150 | 3 | 7 | 6 | 0 | 37 | 51 | 0 | 0 | 46 |
|  | **F5** | 1 | 50 | 0 | 2 | 5 | 0 | 5 | 23 | 0 | 0 | 15 |
|  |  | 2 | 50 | 0 | 3 | 3 | 0 | 3 | 27 | 0 | 0 | 14 |
|  |  | 3 | 50 | 0 | 2 | 6 | 0 | 3 | 27 | 0 | 0 | 12 |
|  |  | Total | 150 | 0 | 7 | 14 | 0 | 11 | 77 | 0 | 0 | 41 |
|  | **F6** | 1 | 50 | 1 | 2 | 7 | 0 | 9 | 16 | 0 | 0 | 15 |
|  |  | 2 | 50 | 0 | 2 | 3 | 0 | 10 | 21 | 0 | 0 | 14 |
|  |  | 3 | 50 | 0 | 3 | 7 | 0 | 7 | 12 | 0 | 0 | 21 |
|  |  | Total | 150 | 1 | 7 | 17 | 0 | 26 | 49 | 0 | 0 | 50 |
|  | **F7** | 1 | 50 | 1 | 12 | 3 | 0 | 7 | 19 | 0 | 0 | 8 |
|  |  | 2 | 50 | 1 | 9 | 3 | 0 | 11 | 14 | 0 | 0 | 12 |
|  |  | 3 | 50 | 1 | 11 | 2 | 0 | 10 | 12 | 0 | 0 | 14 |
|  |  | Total | 150 | 3 | 32 | 8 | 0 | 28 | 45 | 0 | 0 | 34 |
|  | **F8** | 1 | 50 | 2 | 13 | 2 | 0 | 6 | 16 | 0 | 0 | 11 |
|  |  | 2 | 50 | 1 | 8 | 5 | 0 | 10 | 21 | 0 | 0 | 5 |

Continued

Table 1. (Continued)

| **Site** | **Generation** | **Rep** | **N** | **1,534/1,016 di-locus genotype** | | | | | | | | |
| --- | --- | --- | --- | --- | --- | --- | --- | --- | --- | --- | --- | --- |
|  |  |  |  | **VV/FF** | **VV/FC** | **VV/CC** | **IV/FF** | **IV/FC** | **IV/CC** | **II/FF** | **II/FC** | **II/CC** |
|  |  | 3 | 50 | 1 | 10 | 3 | 0 | 11 | 20 | 1 | 0 | 4 |
|  |  | Total | 150 | 4 | 31 | 10 | 0 | 27 | 57 | 1 | 0 | 20 |
