# Supplementary material for: Loss of pyrethroid resistance in newly established laboratory colonies of Aedes aegypti
Source: PLoS Negl Trop Dis. 2020 Mar 16;14(3):e0007753. doi: 10.1371/journal.pntd.0007753 (PMC7117762; doi:10.1371/journal.pntd.0007753)
Supplement: S3 Table — High-density intervals (HDI) 95% were calculated for each biological replicate separately. (DOCX) [file pntd.0007753.s003.docx]

| **Site** | **Generation** | **Rep.** | **I1,016** | **Prob.** | **95% HDI** | **C1,534** | **Prob.** | **95% HDI** |
| --- | --- | --- | --- | --- | --- | --- | --- | --- |
| Acp |  |  | Frequency |  |  | Frequency |  |  |
|  | F1 | 1 | 0.88 |  | (0.7615-0.9428) | 0.98 |  | (0.8953-0.9952) |
|  |  | 2 | 0.84 |  | (0.7143-0.9157) | 1 |  | (0.9302-0.9995) |
|  |  | 3 | 0.78 |  | (0.6532-0.8744) | 1 |  | (0.9316-0.9995) |
|  |  | Total | 0.83 | 0.43 | (0.7883-0.872) | 0.99 | 0.362 | (0.9764-0.998) |
|  | F2 | 1 | 0.77 |  | (0.647-0.8718) | 0.98 |  | (0.8953-0.9952) |
|  |  | 2 | 0.69 |  | (0.5616-0.8088) | 0.94 |  | (0.8379-0.9782) |
|  |  | 3 | 0.77 |  | (0.647-0.8718) | 0.98 |  | (0.8953-0.9952) |
|  |  | Total | 0.74 | 0.563 | (0.691-0.7893) | 0.97 | 0.437 | (0.9398-0.9816) |
|  | F3 | 1 | 0.78 |  | (0.6532-0.8744) | 1 |  | (0.9302-0.9995) |
|  |  | 2 | 0.71 |  | (0.5826-0.825) | 0.96 |  | (0.8655-0.9877) |
|  |  | 3 | 0.67 |  | (0.541-0.7924) | 0.88 |  | (0.7615-0.9428) |
|  |  | Total | 0.72 | 0.529 | (0.6667-0.7677) | 0.95 | 0.025 | (0.9152-0.9667) |
|  | F4 | 1 | 0.66 |  | (0.5206-0.7756) | 0.96 |  | (0.8655-0.9877) |
|  |  | 2 | 0.71 |  | (0.5826-0.825) | 0.9 |  | (0.7859-0.9554) |
|  |  | 3 | 0.66 |  | (0.5206-0.7756) | 0.93 |  | (0.8379-0.9782) |
|  |  | Total | 0.677 | 0.759 | (0.6217-0.727) | 0.93 | 0.472 | (0.8953-0.9536) |
|  | F5 | 1 | 0.68 |  | (0.541-0.7924) | 0.92 |  | (0.8115-0.9673) |
|  |  | 2 | 0.68 |  | (0.541-0.7924) | 0.93 |  | (0.8379-0.9782) |
|  |  | 3 | 0.71 |  | (0.5826-0.825) | 0.92 |  | (0.8115-0.9673) |
|  |  | Total | 0.69 | 0.882 | (0.6355-0.7396) | 0.92 | 0.907 | (0.8876-0.9483) |
|  | F6 | 1 | 0.63 |  | (0.5006-0.7586) | 0.79 |  | (0.669-0.8869) |
|  |  | 2 | 0.69 |  | (0.5616-0.8088) | 0.87 |  | (0.7615-0.9428) |
|  |  | 3 | 0.55 |  | (0.4222-0.6885) | 0.89 |  | (0.7859-0.9554) |
|  |  | Total | 0.62 | 0.346 | (0.5672-0.6762) | 0.85 | 0.313 | (0.8051-0.8859) |
|  | F7 | 1 | 0.49 |  | (0.3658-0.634) | 0.49 |  | (0.3658-0.634) |
|  |  | 2 | 0.47 |  | (0.3476-0.6156) | 0.84 |  | (0.7143-0.9157) |
|  |  | 3 | 0.47 |  | (0.3476-0.6156) | 0.53 |  | (0.4032-0.6706) |
|  |  | Total | 0.48 | 0.974 | (0.4207-0.5332) | 0.62 | 0.001 | (0.5638-0.673) |
|  | F8 | 1 | 0.32 |  | (0.2077-0.4589) | 0.4 |  | (0.276-0.5391) |
|  |  | 2 | 0.32 |  | (0.2077-0.4589) | 0.45 |  | (0.3295-0.5966) |
|  |  | 3 | 0.28 |  | (0.175-0.4174) | 0.33 |  | (0.2245-0.4793) |
|  |  | Total | 0.31 | 0.882 | (0.2572-0.361) | 0.39 | 0.472 | (0.3398-0.4497) |
| Tap |  |  |  |  |  |  |  |  |
|  | F1 | 1 | 0.61 |  | (0.4806-0.7415) | 1 |  | (0.9302-0.9995) |
|  |  | 2 | 0.56 |  | (0.4222-0.6885) | 1 |  | (0.9302-0.9995) |
|  |  | 3 | 0.65 |  | (0.5206-0.7756) | 0.94 |  | (0.8379-0.9782) |
|  |  | Total | 0.61 | 0.586 | (0.5503-0.6602) | 0.98 | 0.047 | (0.9572-0.9906) |
|  | F2 | 1 | 0.77 |  | (0.647-0.8718) | 1 |  | (0.9302-0.9995) |
|  |  | 2 | 0.71 |  | (0.5826-0.825) | 1 |  | (0.9302-0.9995) |
|  |  | 3 | 0.67 |  | (0.541-0.7924) | 1 |  | (0.9302-0.9995) |
|  |  | Total | 0.72 | 0.529 | (0.6632-0.7646) | 1 | - | (0.9878-0.9999) |
|  | F3 | 1 | 0.6 |  | (0.461-0.724) | 1 |  | (0.9302-0.9995) |
|  |  | 2 | 0.64 |  | (0.5006-0.7586) | 1 |  | (0.9302-0.9995) |
|  |  | 3 | 0.61 |  | (0.4806-0.7415) | 1 |  | (0.9302-0.9995) |
|  |  | Total | 0.617 | 0.919 | (0.5604-0.6698) | 1 | - | (0.9878-0.9999) |
|  | F4 | 1 | 0.62 |  | (0.4806-0.7415) | 1 |  | (0.9302-0.9995) |
|  |  | 2 | 0.6 |  | (0.461-0.724) | 1 |  | (0.9302-0.9995) |
|  |  | 3 | 0.64 |  | (0.5006-0.7586) | 1 |  | (0.9302-0.9995) |
|  |  | Total | 0.62 | 0.919 | (0.5638-0.673) | 1 | - | (0.9878-0.9999) |
|  | F5 | 1 | 0.45 |  | (0.3295-0.5966) | 0.99 |  | (0.9302-0.9995) |
|  |  | 2 | 0.62 |  | (0.4806-0.7415) | 0.98 |  | (0.8953-0.9952) |
|  |  | 3 | 0.47 |  | (0.3476-0.6156) | 0.99 |  | (0.9302-0.9995) |
|  |  | Total | 0.51 | 0.218 | (0.4569-0.5693) | 0.99 | 0.365 | (0.9664-0.9946) |
|  | F6 | 1 | 0.53 |  | (0.4032-0.6706) | 1 |  | (0.9302-0.9995) |
|  |  | 2 | 0.48 |  | (0.3476-0.6156) | 1 |  | (0.9302-0.9995) |
|  |  | 3 | 0.59 |  | (0.461-0.724) | 1 |  | (0.9302-0.9995) |
|  |  | Total | 0.53 | 0.485 | (0.4768-0.5889) | 1 | - | (0.9878-0.9999) |
|  | F7 | 1 | 0.52 |  | (0.3845-0.6522) | 1 |  | (0.9302-0.9995) |
|  |  | 2 | 0.62 |  | (0.4806-0.7415) | 1 |  | (0.9302-0.9995) |
|  |  | 3 | 0.55 |  | (0.4222-0.6885) | 1 |  | (0.9302-0.9995) |
|  |  | Total | 0.56 | 0.597 | (0.5067-0.6183) | 1 | - | (0.9878-0.9999) |
|  | F8 | 1 | 0.59 |  | (0.461-0.724) | 1 |  | (0.9302-0.9995) |
|  |  | 2 | 0.57 |  | (0.4415-0.7062) | 0.99 |  | (0.9302-0.9995) |
|  |  | 3 | 0.6 |  | (0.461-0.724) | 1 |  | (0.9302-0.9995) |
|  |  | Total | 0.59 | 0.973 | (0.5301-0.641) | 1 | - | (0.9816-0.9992) |
| Mer1 |  |  |  |  |  |  |  |  |
|  | F1 | 1 | 0.59 |  | (0.461-0.724) | 0.88 |  | (0.7615-0.9428) |
|  |  | 2 | 0.58 |  | (0.4415-0.7062) | 0.94 |  | (0.8379-0.9782) |
|  |  | 3 | 0.64 |  | (0.4345-0.8369) | 0.92 |  | (0.7392-0.987) |
|  |  | Total | 0.59 | 0.812 | (0.5295-0.6539) | 0.91 | 0.498 | (0.8678-0.9409) |
|  | F2 | 1 | 0.43 |  | (0.3114-0.5777) | 0.92 |  | (0.8115-0.9673) |
|  |  | 2 | 0.44 |  | (0.3114-0.5777) | 0.88 |  | (0.7615-0.9428) |
|  |  | 3 | 0.48 |  | (0.3476-0.6156) | 0.93 |  | (0.8379-0.9782) |
|  |  | Total | 0.45 | 0.898 | (0.3946-0.5066) | 0.91 | 0.555 | (0.8722-0.9373) |
|  | F3 | 1 | 0.47 |  | (0.3476-0.6156) | 0.77 |  | (0.647-0.8718) |
|  |  | 2 | 0.43 |  | (0.3114-0.5777) | 0.95 |  | (0.8655-0.9877) |
|  |  | 3 | 0.45 |  | (0.3295-0.5966) | 0.95 |  | (0.8655-0.9877) |
|  |  | Total | 0.45 | 0.923 | (0.3946-0.5066) | 0.89 | 0.002 | (0.8495-0.9205) |
|  | F4 | 1 | 0.3 |  | (0.1913-0.4383) | 0.94 |  | (0.8379-0.9782) |
|  |  | 2 | 0.47 |  | (0.3476-0.6156) | 0.94 |  | (0.8379-0.9782) |
|  |  | 3 | 0.38 |  | (0.2586-0.5193) | 0.94 |  | (0.8379-0.9782) |
|  |  | Total | 0.38 | 0.18 | (0.3301-0.4395) | 0.94 | 1 | (0.9072-0.9615) |
|  | F5 | 1 | 0.45 |  | (0.3295-0.5966) | 1 |  | (0.9302-0.9995) |
|  |  | 2 | 0.43 |  | (0.3114-0.5777) | 1 |  | (0.9302-0.9995) |
|  |  | 3 | 0.52 |  | (0.3845-0.6522) | 0.97 |  | (0.8953-0.9952) |
|  |  | Total | 0.47 | 0.706 | (0.4109-0.5233) | 0.99 | 0.365 | (0.9712-0.9964) |
|  | F6 | 1 | 0.49 |  | (0.3658-0.634) | 0.99 |  | (0.9302-0.9995) |
|  |  | 2 | 0.46 |  | (0.3295-0.5966) | 0.96 |  | (0.8655-0.9877) |
|  |  | 3 | 0.53 |  | (0.4032-0.6706) | 0.98 |  | (0.8953-0.9952) |
|  |  | Total | 0.49 | 0.726 | (0.4372-0.5497) | 0.98 | 0.36 | (0.9527-0.9884) |
|  | F7 | 1 | 0.46 |  | (0.3295-0.5966) | 0.95 |  | (0.8655-0.9877) |
|  |  | 2 | 0.32 |  | (0.2077-0.4589) | 0.94 |  | (0.8379-0.9782) |
|  |  | 3 | 0.41 |  | (0.2937-0.5587) | 0.99 |  | (0.9302-0.9995) |
|  |  | Total | 0.4 | 0.339 | (0.343-0.4531) | 0.96 | 0.235 | (0.9315-0.9768) |
|  | F8 | 1 | 0.23 |  | (0.1435-0.3748) | 0.94 |  | (0.8379-0.9782) |
|  |  | 2 | 0.45 |  | (0.3295-0.5966) | 0.98 |  | (0.8953-0.9952) |
|  |  | 3 | 0.35 |  | (0.2414-0.4994) | 0.97 |  | (0.8953-0.9952) |
|  |  | Total | 0.34 | 0.07 | (0.2919-0.3988) | 0.96 | 0.437 | (0.9356-0.9792) |
| Mer2 |  |  |  |  |  |  |  |  |
|  | F1 | 1 | 0.54 |  | (0.4032-0.6706) | 0.84 |  | (0.7143-0.9157) |
|  |  | 2 | 0.58 |  | (0.4415-0.7062) | 0.85 |  | (0.7377-0.9296) |
|  |  | 3 | 0.64 |  | (0.5006-0.7586) | 0.91 |  | (0.8115-0.9673) |
|  |  | Total | 0.59 | 0.593 | (0.5301-0.641) | 0.87 | 0.457 | (0.8235-0.9005) |
|  | F2 | 1 | 0.46 |  | (0.3295-0.5966) | 0.81 |  | (0.6915-0.9015) |
|  |  | 2 | 0.45 |  | (0.3295-0.5966) | 0.8 |  | (0.669-0.8869) |
|  |  | 3 | 0.35 |  | (0.2414-0.4994) | 0.7 |  | (0.5616-0.8088) |
|  |  | Total | 0.42 | 0.506 | (0.3655-0.4765) | 0.77 | 0.308 | (0.7191-0.814) |
|  | F3 | 1 | 0.43 |  | (0.3114-0.5777) | 0.78 |  | (0.647-0.8718) |
|  |  | 2 | 0.36 |  | (0.2414-0.4994) | 0.68 |  | (0.541-0.7924) |
|  |  | 3 | 0.42 |  | (0.2937-0.5587) | 0.73 |  | (0.6037-0.8409) |
|  |  | Total | 0.4 | 0.698 | (0.3494-0.4598) | 0.73 | 0.523 | (0.6771-0.777) |
|  | F4 | 1 | 0.38 |  | (0.2586-0.5193) | 0.79 |  | (0.669-0.8869) |
|  |  | 2 | 0.4 |  | (0.276-0.5391) | 0.77 |  | (0.647-0.8718) |
|  |  | 3 | 0.33 |  | (0.2245-0.4793) | 0.79 |  | (0.669-0.8869) |
|  |  | Total | 0.37 | 0.819 | (0.3173-0.426) | 0.78 | 0.96 | (0.733-0.8261) |
|  | F5 | 1 | 0.38 |  | (0.2586-0.5193) | 0.58 |  | (0.4415-0.7062) |
|  |  | 2 | 0.37 |  | (0.2586-0.5193) | 0.56 |  | (0.4222-0.6885) |
|  |  | 3 | 0.34 |  | (0.2245-0.4793) | 0.46 |  | (0.3295-0.5966) |
|  |  | Total | 0.36 | 0.892 | (0.311-0.4193) | 0.53 | 0.436 | (0.4768-0.5889) |
|  | F6 | 1 | 0.21 |  | (0.1283-0.3531) | 0.33 |  | (0.2245-0.4793) |
|  |  | 2 | 0.37 |  | (0.2586-0.5193) | 0.52 |  | (0.3845-0.6522) |
|  |  | 3 | 0.3 |  | (0.1913-0.4383) | 0.47 |  | (0.3476-0.6156) |
|  |  | Total | 0.29 | 0.218 | (0.2447-0.3472) | 0.44 | 0.164 | (0.3849-0.4966) |
|  | F7 | 1 | 0.31 |  | (0.2077-0.4589) | 0.45 |  | (0.3295-0.5966) |
|  |  | 2 | 0.28 |  | (0.175-0.4174) | 0.49 |  | (0.3658-0.634) |
|  |  | 3 | 0.35 |  | (0.2414-0.4994) | 0.56 |  | (0.4222-0.6885) |
|  |  | Total | 0.31 | 0.692 | (0.2634-0.3679) | 0.5 | 0.602 | (0.4437-0.5562) |
|  | F8 | 1 | 0.25 |  | (0.1592-0.3964) | 0.52 |  | (0.3845-0.6522) |
|  |  | 2 | 0.37 |  | (0.2586-0.5193) | 0.49 |  | (0.3658-0.634) |
|  |  | 3 | 0.33 |  | (0.2189-0.4896) | 0.52 |  | (0.3785-0.6627) |
|  |  | Total | 0.32 | 0.428 | (0.265-0.3718) | 0.51 | 0.971 | (0.4529-0.5676) |
| Mer3 |  |  |  |  |  |  |  |  |
|  | F1 | 1 | 0.63 |  | (0.5006-0.7586) | 1 |  | (0.9302-0.9995) |
|  |  | 2 | 0.81 |  | (0.6915-0.9015) | 1 |  | (0.9302-0.9995) |
|  |  | 3 | 0.75 |  | (0.6253-0.8565) | 1 |  | (0.9302-0.9995) |
|  |  | Total | 0.73 | 0.113 | (0.6771-0.777) | 1 | - | (0.9878-0.9999) |
|  | F2 | 1 | 0.81 |  | (0.6915-0.9015) | 1 |  | (0.9302-0.9995) |
|  |  | 2 | 0.72 |  | (0.5826-0.825) | 0.98 |  | (0.8953-0.9952) |
|  |  | 3 | 0.78 |  | (0.647-0.8718) | 1 |  | (0.9302-0.9995) |
|  |  | Total | 0.77 | 0.486 | (0.7191-0.814) | 0.99 | 0.365 | (0.9762-0.9979) |
|  | F3 | 1 | 0.63 |  | (0.5006-0.7586) | 0.9 |  | (0.7859-0.9554) |
|  |  | 2 | 0.74 |  | (0.6037-0.8409) | 0.98 |  | (0.8953-0.9952) |
|  |  | 3 | 0.8 |  | (0.669-0.8869) | 1 |  | (0.9302-0.9995) |
|  |  | Total | 0.72 | 0.193 | (0.6701-0.7708) | 0.96 | 0.026 | (0.9315-0.9768) |
|  | F4 | 1 | 0.83 |  | (0.7143-0.9157) | 1 |  | (0.9302-0.9995) |
|  |  | 2 | 0.63 |  | (0.5006-0.7586) | 0.99 |  | (0.9302-0.9995) |
|  |  | 3 | 0.67 |  | (0.541-0.7924) | 0.82 |  | (0.6915-0.9015) |
|  |  | Total | 0.71 | 0.062 | (0.6562-0.7583) | 0.94 | 0 | (0.9032-0.9589) |
|  | F5 | 1 | 0.41 |  | (0.2937-0.5587) | 0.99 |  | (0.9302-0.9995) |
|  |  | 2 | 0.52 |  | (0.3845-0.6522) | 1 |  | (0.9302-0.9995) |
|  |  | 3 | 0.4 |  | (0.276-0.5391) | 1 |  | (0.9302-0.9995) |
|  |  | Total | 0.44 | 0.433 | (0.3881-0.5) | 1 | - | (0.9816-0.9992) |
|  | F6 | 1 | 0.44 |  | (0.3114-0.5777) | 1 |  | (0.9302-0.9995) |
|  |  | 2 | 0.48 |  | (0.3476-0.6156) | 1 |  | (0.9302-0.9995) |
|  |  | 3 | 0.46 |  | (0.3295-0.5966) | 1 |  | (0.9302-0.9995) |
|  |  | Total | 0.46 | 0.923 | (0.4044-0.5166) | 1 | - | (0.9878-0.9999) |
|  | F7 | 1 | 0.38 |  | (0.2586-0.5193) | 1 |  | (0.9302-0.9995) |
|  |  | 2 | 0.47 |  | (0.3476-0.6156) | 0.99 |  | (0.9302-0.9995) |
|  |  | 3 | 0.65 |  | (0.5206-0.7756) | 1 |  | (0.9302-0.9995) |
|  |  | Total | 0.5 | 0.018 | (0.4437-0.5562) | 1 | - | (0.9816-0.9992) |
|  | F8 | 1 | 0.63 |  | (0.5006-0.7586) | 1 |  | (0.9302-0.9995) |
|  |  | 2 | 0.63 |  | (0.5006-0.7586) | 0.99 |  | (0.9302-0.9995) |
|  |  | 3 | 0.64 |  | (0.5006-0.7586) | 1 |  | (0.9302-0.9995) |
|  |  | Total | 0.63 | 1 | (0.5773-0.6858) | 1 | - | (0.9816-0.9992) |
| Dz |  |  |  |  |  |  |  |  |
|  | F1 | 1 | 0.37 |  | (0.2586-0.5193) | 0.71 |  | (0.5826-0.825) |
|  |  | 2 | 0.29 |  | (0.1913-0.4383) | 0.62 |  | (0.4806-0.7415) |
|  |  | 3 | 0.39 |  | (0.276-0.5391) | 0.69 |  | (0.5616-0.8088) |
|  |  | Total | 0.35 | 0.545 | (0.2982-0.4056) | 0.67 | 0.526 | (0.6183-0.7239) |
|  | F2 | 1 | 0.7 |  | (0.5616-0.8088) | 0.98 |  | (0.8953-0.9952) |
|  |  | 2 | 0.58 |  | (0.4415-0.7062) | 0.86 |  | (0.7377-0.9296) |
|  |  | 3 | 0.37 |  | (0.2586-0.5193) | 0.71 |  | (0.5826-0.825) |
|  |  | Total | 0.55 | 0.005 | (0.4933-0.6053) | 0.85 | 0.001 | (0.8051-0.8859) |
|  | F3 | 1 | 0.49 |  | (0.3658-0.634) | 0.67 |  | (0.541-0.7924) |
|  |  | 2 | 0.37 |  | (0.2586-0.5193) | 0.62 |  | (0.4806-0.7415) |
|  |  | 3 | 0.4 |  | (0.276-0.5391) | 0.72 |  | (0.5826-0.825) |
|  |  | Total | 0.42 | 0.43 | (0.3655-0.4765) | 0.67 | 0.562 | (0.6149-0.7207) |
|  | F4 | 1 | 0.4 |  | (0.276-0.5391) | 0.52 |  | (0.3845-0.6522) |
|  |  | 2 | 0.41 |  | (0.2937-0.5587) | 0.7 |  | (0.5616-0.8088) |
|  |  | 3 | 0.58 |  | (0.4415-0.7062) | 0.77 |  | (0.647-0.8718) |
|  |  | Total | 0.463 | 0.142 | (0.4077-0.52) | 0.66 | 0.019 | (0.608-0.7144) |
|  | F5 | 1 | 0.42 |  | (0.2937-0.5587) | 0.79 |  | (0.669-0.8869) |
|  |  | 2 | 0.44 |  | (0.3114-0.5777) | 0.79 |  | (0.669-0.8869) |
|  |  | 3 | 0.46 |  | (0.3295-0.5966) | 0.74 |  | (0.6037-0.8409) |
|  |  | Total | 0.44 | 0.922 | (0.3849-0.4966) | 0.77 | 0.705 | (0.7226-0.817) |
|  | F6 | 1 | 0.47 |  | (0.3476-0.6156) | 0.74 |  | (0.6037-0.8409) |
|  |  | 2 | 0.48 |  | (0.3476-0.6156) | 0.68 |  | (0.541-0.7924) |
|  |  | 3 | 0.45 |  | (0.3295-0.5966) | 0.69 |  | (0.5616-0.8088) |
|  |  | Total | 0.467 | 0.974 | (0.4109-0.5233) | 0.7 | 0.798 | (0.6493-0.7521) |
|  | F7 | 1 | 0.36 |  | (0.2414-0.4995) | 0.68 |  | (0.541-0.7924) |
|  |  | 2 | 0.37 |  | (0.2586-0.5193) | 0.6 |  | (0.461-0.724) |
|  |  | 3 | 0.42 |  | (0.2937-0.5587) | 0.65 |  | (0.5206-0.7756) |
|  |  | Total | 0.383 | 0.821 | (0.3301-0.4395) | 0.64 | 0.684 | (0.5875-0.6953) |
|  | F8 | 1 | 0.3 |  | (0.1913-0.4383) | 0.42 |  | (0.2937-0.5587) |
|  |  | 2 | 0.29 |  | (0.1913-0.4383) | 0.58 |  | (0.4415-0.7062) |
|  |  | 3 | 0.39 |  | (0.276-0.5391) | 0.74 |  | (0.6037-0.8409) |
|  |  | Total | 0.327 | 0.472 | (0.2761-0.3816) | 0.58 | 0.005 | (0.5234-0.6345) |
| Co |  |  |  |  |  |  |  |  |
|  | F1 | 1 | 0.69 |  | (0.5616-0.8088) | 0.88 |  | (0.7615-0.9428) |
|  |  | 2 | 0.48 |  | (0.3476-0.6156) | 0.57 |  | (0.4415-0.7062) |
|  |  | 3 | 0.65 |  | (0.5206-0.7756) | 0.8 |  | (0.669-0.8869) |
|  |  | Total | 0.607 | 0.055 | (0.5503-0.6602) | 0.75 | 0.002 | (0.698-0.7955) |
|  | F2 | 1 | 0.84 |  | (0.7143-0.9157) | 0.92 |  | (0.8115-0.9673) |
|  |  | 2 | 0.57 |  | (0.4415-0.7062) | 0.76 |  | (0.6253-0.8565) |
|  |  | 3 | 0.46 |  | (0.3295-0.5966) | 0.7 |  | (0.5616-0.8088) |
|  |  | Total | 0.623 | 0 | (0.5672-0.6762) | 0.79 | 0.019 | (0.7439-0.8352) |
|  | F3 | 1 | 0.51 |  | (0.3845-0.6522) | 0.63 |  | (0.5006-0.7586) |
|  |  | 2 | 0.44 |  | (0.3114-0.5777) | 0.68 |  | (0.541-0.7924) |
|  |  | 3 | 0.48 |  | (0.3476-0.6156) | 0.69 |  | (0.5616-0.8088) |
|  |  | Total | 0.477 | 0.726 | (0.4207-0.5332) | 0.67 | 0.809 | (0.6114-0.7175) |
|  | F4 | 1 | 0.46 |  | (0.3295-0.5966) | 0.72 |  | (0.5826-0.825) |
|  |  | 2 | 0.45 |  | (0.3295-0.5966) | 0.65 |  | (0.5206-0.7756) |
|  |  | 3 | 0.47 |  | (0.3476-0.6156) | 0.59 |  | (0.461-0.724) |
|  |  | Total | 0.46 | 0.974 | (0.4044-0.5166) | 0.65 | 0.448 | (0.5978-0.7049) |
|  | F5 | 1 | 0.18 |  | (0.09863-0.3085) | 0.28 |  | (0.175-0.4174) |
|  |  | 2 | 0.16 |  | (0.08439-0.2857) | 0.3 |  | (0.1913-0.4383) |
|  |  | 3 | 0.25 |  | (0.1592-0.3964) | 0.38 |  | (0.2586-0.5193) |
|  |  | Total | 0.197 | 0.417 | (0.1557-0.2454) | 0.32 | 0.526 | (0.2698-0.3748) |
|  | F6 | 1 | 0.06 |  | (0.02176-0.1621) | 0.27 |  | (0.175-0.4174) |
|  |  | 2 | 0.07 |  | (0.03267-0.1885) | 0.28 |  | (0.175-0.4174) |
|  |  | 3 | 0.09 |  | (0.0446-0.2138) | 0.26 |  | (0.1592-0.3964) |
|  |  | Total | 0.073 | 0.762 | (0.04908-0.1086) | 0.27 | 0.967 | (0.2229-0.3229) |
|  | F7 | 1 | 0.18 |  | (0.09863-0.3085) | 0.41 |  | (0.2937-0.5587) |
|  |  | 2 | 0.1 |  | (0.0446-0.2138) | 0.18 |  | (0.09863-0.3085) |
|  |  | 3 | 0.08 |  | (0.03267-0.1885) | 0.12 |  | (0.05727-0.2383) |
|  |  | Total | 0.12 | 0.266 | (0.08801-0.1617) | 0.24 | 0.001 | (0.1921-0.2879) |
|  | F8 | 1 | 0.6 |  | (0.461-0.724) | 0.16 |  | (0.08439-0.2857) |
|  |  | 2 | 0.31 |  | (0.2077-0.4589) | 0.09 |  | (0.0446-0.2138) |
|  |  | 3 | 0.37 |  | (0.2586-0.5193) | 0.62 |  | (0.4806-0.7415) |
|  |  | Total | 0.427 | 0.012 | (0.3719-0.4832) | 0.29 | 0 | (0.2416-0.3439) |
| Ac |  |  |  |  |  |  |  |  |
|  | F1 | 1 | 0.79 |  | (0.6532-0.8744) | 0.94 |  | (0.8379-0.9782) |
|  |  | 2 | 0.78 |  | (0.647-0.8718) | 0.92 |  | (0.8115-0.9673) |
|  |  | 3 | 0.74 |  | (0.6037-0.8409) | 0.89 |  | (0.7859-0.9554) |
|  |  | Total | 0.77 | 0.766 | (0.7191-0.814) | 0.92 | 0.762 | (0.8799-0.9428) |
|  | F2 | 1 | 0.54 |  | (0.4032-0.6706) | 0.66 |  | (0.5206-0.7756) |
|  |  | 2 | 0.74 |  | (0.6037-0.8409) | 0.9 |  | (0.7859-0.9554) |
|  |  | 3 | 0.8 |  | (0.6532-0.8744) | 0.91 |  | (0.8115-0.9673) |
|  |  | Total | 0.693 | 0.013 | (0.6389-0.7427) | 0.82 | 0.001 | (0.7761-0.8623) |
|  | F3 | 1 | 0.55 |  | (0.4222-0.6885) | 0.71 |  | (0.5826-0.825) |
|  |  | 2 | 0.74 |  | (0.6037-0.8409) | 0.85 |  | (0.7377-0.9296) |
|  |  | 3 | 0.74 |  | (0.6037-0.8409) | 0.9 |  | (0.7859-0.9554) |
|  |  | Total | 0.677 | 0.084 | (0.6217-0.727) | 0.82 | 0.044 | (0.7725-0.8593) |
|  | F4 | 1 | 0.6 |  | (0.461-0.724) | 0.81 |  | (0.6915-0.9015) |
|  |  | 2 | 0.64 |  | (0.5006-0.7586) | 0.86 |  | (0.7377-0.9296) |
|  |  | 3 | 0.56 |  | (0.4222-0.6885) | 0.83 |  | (0.7143-0.9157) |
|  |  | Total | 0.6 | 0.717 | (0.5435-0.6538) | 0.83 | 0.862 | (0.7869-0.8712) |
|  | F5 | 1 | 0.58 |  | (0.4415-0.7062) | 0.93 |  | (0.8379-0.9782) |
|  |  | 2 | 0.58 |  | (0.4415-0.7062) | 0.94 |  | (0.8379-0.9782) |
|  |  | 3 | 0.54 |  | (0.4032-0.6706) | 0.95 |  | (0.8655-0.9877) |
|  |  | Total | 0.567 | 0.897 | (0.51-0.6215) | 0.94 | 0.876 | (0.9072-0.9615) |
|  | F6 | 1 | 0.55 |  | (0.4222-0.6885) | 0.87 |  | (0.7615-0.9428) |
|  |  | 2 | 0.59 |  | (0.461-0.724) | 0.88 |  | (0.7615-0.9428) |
|  |  | 3 | 0.61 |  | (0.4806-0.7415) | 0.9 |  | (0.7859-0.9554) |
|  |  | Total | 0.583 | 0.824 | (0.5268-0.6378) | 0.88 | 0.936 | (0.842-0.9148) |
|  | F7 | 1 | 0.42 |  | (0.2937-0.5587) | 0.79 |  | (0.669-0.8869) |
|  |  | 2 | 0.49 |  | (0.3658-0.634) | 0.78 |  | (0.647-0.8718) |
|  |  | 3 | 0.5 |  | (0.3658-0.634) | 0.77 |  | (0.647-0.8718) |
|  |  | Total | 0.47 | 0.652 | (0.4142-0.5265) | 0.78 | 0.961 | (0.7297-0.8231) |
|  | F8 | 1 | 0.44 |  | (0.3114-0.5777) | 0.77 |  | (0.647-0.8718) |
|  |  | 2 | 0.41 |  | (0.2937-0.5587) | 0.8 |  | (0.669-0.8869) |
|  |  | 3 | 0.41 |  | (0.2937-0.5587) | 0.75 |  | (0.6253-0.8565) |
|  |  | Total | 0.42 | 0.973 | (0.3655-0.4765) | 0.77 | 0.89 | (0.7226-0.817) |
